# Performance degradation and failure mechanisms of pultruded carbon/glass-fiber hybrid sucker rods after long-term downhole service

**DOI:** 10.1371/journal.pone.0360408

**Published:** 2026-10-15

**Authors:** Dongxu He, Tao Jiang, Huajing Xu

**Affiliations:** 1 School of Mechanical Engineering, Yangtze University, Jingzhou, Hubei, China; 2 Production Plant, Shengli Oilfield Branch, China Petroleum & Chemical Corporation, Dongying, Shandong, China; King Mongkut’s University of Technology North Bangkok, THAILAND

## Abstract

Pultruded carbon/glass-fiber hybrid composite rods are increasingly used as sucker rods, yet the relationship between degradation accumulated during long-term downhole service and subsequent longitudinal splitting during retrieval and drum winding remains insufficiently understood, particularly for rods containing a carbon/glass hybrid transition region. This study compared five specimens sectioned from a pristine 22 mm-diameter parent rod with five specimens sectioned from a service-aged parent rod retrieved after 435 days of actual downhole service. Tensile, compressive, flexural, and apparent horizontal shear tests were combined with Welch’s t-tests, bootstrap-assisted two-parameter Weibull analysis, scanning electron microscopy, dynamic mechanical analysis, and moisture-desorption measurements to characterize mechanical degradation and matrix/interfacial damage. After service, the mean tensile, flexural, and apparent short-beam shear strengths decreased by 10.02%, 19.92%, and 21.98%, respectively. After Holm correction, the reductions in flexural and apparent short-beam shear strength remained statistically significant, whereas the tensile-strength reduction did not (adjusted p = 0.057). The mean compressive strength decreased by only 2.25% and showed no statistically significant difference; however, its coefficient of variation increased from 2.42% to 18.88%, while the Weibull shape-parameter estimate decreased from 41.31 to 5.27, suggesting greater compressive-strength dispersion. For the carbon-fiber core, the storage modulus decreased by 34.3%, whereas the glass-transition temperature increased from 159.5 to 164.76 °C. These changes may reflect competing contributions from residual post-curing and moisture-related effects, although the underlying chemical mechanisms were not directly characterized. The greater equilibrium mass loss of the service-aged specimens during drying was consistent with greater retention of removable moisture. Scanning electron microscopy observations revealed extensive matrix fragmentation, resin loss, fiber–matrix debonding, and longitudinal interfacial cracking. Collectively, the results support a possible two-phase damage-evolution process in which prolonged fluid exposure and repeated operational loading progressively weaken the matrix and hybrid interfaces, while additional bending during retrieval and drum winding may promote the propagation and coalescence of pre-existing cracks, thereby contributing to local longitudinal splitting. These findings provide insight into the relationships among field exposure, loading-mode-dependent degradation, strength dispersion, and interfacial damage in three-region hybrid composite sucker rods.

## 1. Introduction

With the continued growth in global energy demand, oil production is increasingly extending to challenging operating conditions, including deep and ultra-deep wells, highly deviated wells, and corrosive environments [1,2]. In rod-pumping systems, the sucker-rod string connects the surface pumping unit to the downhole pump and therefore plays an important role in operational efficiency, energy consumption, and service life [3]. Although conventional steel sucker rods are technologically mature and relatively inexpensive, their high density, high elastic modulus, and susceptibility to corrosion can become disadvantages under deep-pumping, corrosive, and high-frequency operating conditions [4,5]. Pultruded carbon/glass-fiber-reinforced composite (PFRC) sucker rods have attracted increasing attention because of their low density, high strength, corrosion resistance, and fatigue resistance, offering potential advantages in reducing energy consumption and extending service life [6,7]. During normal downhole operation, PFRC sucker rods are subjected predominantly to cyclic axial tensile and compressive loading generated by reciprocating pumping. Additional bending deformation can occur during retrieval and drum winding. Over prolonged service, the accumulation of matrix cracking, fiber fracture, and interfacial debonding may progressively degrade the mechanical performance of the composite and increase the risk of structural damage [8].

Previous studies have extensively examined how hybrid architecture affects the mechanical response of carbon/glass-fiber composite rods. Xian et al. investigated different carbon/glass hybrid configurations through experimental and theoretical analyses and reported that a uniformly dispersed hybrid architecture exhibited improved mechanical performance and pseudo-ductility by reducing the influence of weak interfacial layers [9]. To mitigate interlaminar splitting, Zhang et al. proposed an intra-ply carbon/glass hybrid rod and reported, on the basis of numerical analysis, a 14.0% increase in ultimate tensile load compared with an inter-ply configuration [10]. Guo et al. further investigated the tension–tension fatigue behavior of pultruded carbon/glass-fiber composite rods and found that the uniformly dispersed hybrid mode exhibited better fatigue performance than the shell–core hybrid configuration [11]. Shafiei et al. combined multiscale modeling with experiments to investigate hybrid carbon/glass textile-reinforced composites and demonstrated that optimization of the ply stacking sequence could improve mechanical performance and energy-absorption capacity [12]. Environmental durability and aging behavior have also received considerable attention. Li et al. investigated the evolution of mechanical properties and service-life prediction of carbon/glass hybrid rods under oil-well service and simulated coupled environmental conditions [13–15]. Xian et al. examined the durability and mechanical behavior of carbon/glass hybrid rods under different temperature and environmental conditions [16–19]. Pan et al. studied the durability of carbon/glass hybrid rods in alkaline environments and reported changes in the durability behavior of the GFRP constituents under alkaline exposure [20,21]. Da Silva et al. investigated the accelerated aging of pultruded CFRP rods manufactured with epoxy and vinyl-ester matrices and found that the vinyl-ester-based rods showed greater sensitivity to fiber–matrix interfacial degradation in short-beam testing [22]. Gao et al. examined the effects of steel-fiber content and carbon/glass-fiber ratio on the interlaminar shear performance of steel–carbon/glass hybrid composite reinforcement and reported that both hybrid composition and elevated temperature affected interlaminar shear performance [23]. Other studies have addressed additional material and structural factors governing the performance of hybrid composites. Recent studies have also shown that constituent selection and hybrid reinforcement design can markedly influence the tensile, flexural, and impact performance of fiber-reinforced composites [24]. Dahil et al. investigated the tensile strength and impact toughness of inter-ply carbon/glass composites with and without surface cracks and reported improvements in both properties with increasing ply number [25]. Waters et al. examined the effects of fiber misalignment on the flexural response of pultruded hybrid composite rods and showed that misalignment reduced flexural strength, whereas superimposed axial tension could mitigate this effect [26]. Naito investigated the static and fatigue bending behavior of carbon/glass hybrid thermoplastic-epoxy composite rods and reported that increasing void content reduced bending strength, while improved static strength was associated with better fatigue performance [27,28]. Zhang et al. employed a genetic algorithm–backpropagation model to evaluate the influence of material design parameters on tensile strength and identified the glass-fiber volume fraction as an important design variable [29].

In summary, previous studies have extensively investigated the effects of hybrid architecture, fatigue loading, elevated temperature, moisture exposure, and simulated oil-well environments on the mechanical properties and durability of carbon/glass hybrid composite rods. Several field-service studies have also reported changes in strength retention and interfacial performance. However, the available investigations have mainly focused on individual mechanical properties, accelerated aging behavior, or service-life prediction. The relationship between actual field-observed longitudinal splitting and the coupled evolution of mechanical-property dispersion, moisture retention, matrix thermomechanical degradation, and fiber–matrix interfacial damage remains insufficiently clarified. In particular, limited information is available on the degradation and failure behavior of field-retrieved PFRC sucker rods with a three-region radial architecture consisting of an outer glass-fiber layer, an intermediate carbon/glass hybrid transition region, and an inner carbon-fiber core.

Therefore, the present study investigates the degradation and failure behavior of the PFRC sucker rod after 435 days of actual downhole service. The scientific contributions of this study are as follows: (a) the field-retrieved specimens provide evidence of loading-mode-dependent changes in both strength loss and strength variability; (b) pronounced matrix and interfacial damage in the carbon/glass hybrid transition region is experimentally identified and is associated with the comparatively greater reductions in flexural and apparent short-beam shear performance; and (c) a possible two-stage damage-evolution framework is proposed, in which cumulative matrix and interfacial degradation may have developed during downhole service, while additional bending during retrieval and drum winding may have promoted the propagation and coalescence of pre-existing cracks.

## 2. Materials and methods

### 2.1. Field fracture failure

During a scheduled pump-inspection operation, the PFRC rod string was retrieved from Well Yan 22−2. A local longitudinal splitting failure occurred during the subsequent retrieval and drum-winding process, as shown in Fig 1. The affected region exhibited pronounced splitting along the rod axis, accompanied by opening of the rod body and local exposure of the internal fiber-reinforced structure. The investigated rod section originated from a depth of approximately 1000–1020 m, corresponding to a nearly vertical interval of the wellbore. Field access and retrieval of the investigated PFRC rod were authorized by Production Plant, Shengli Oilfield Branch, China Petroleum & Chemical Corporation. No additional research permit was required because the specimens were obtained during a scheduled production maintenance operation.

**Fig 1:**
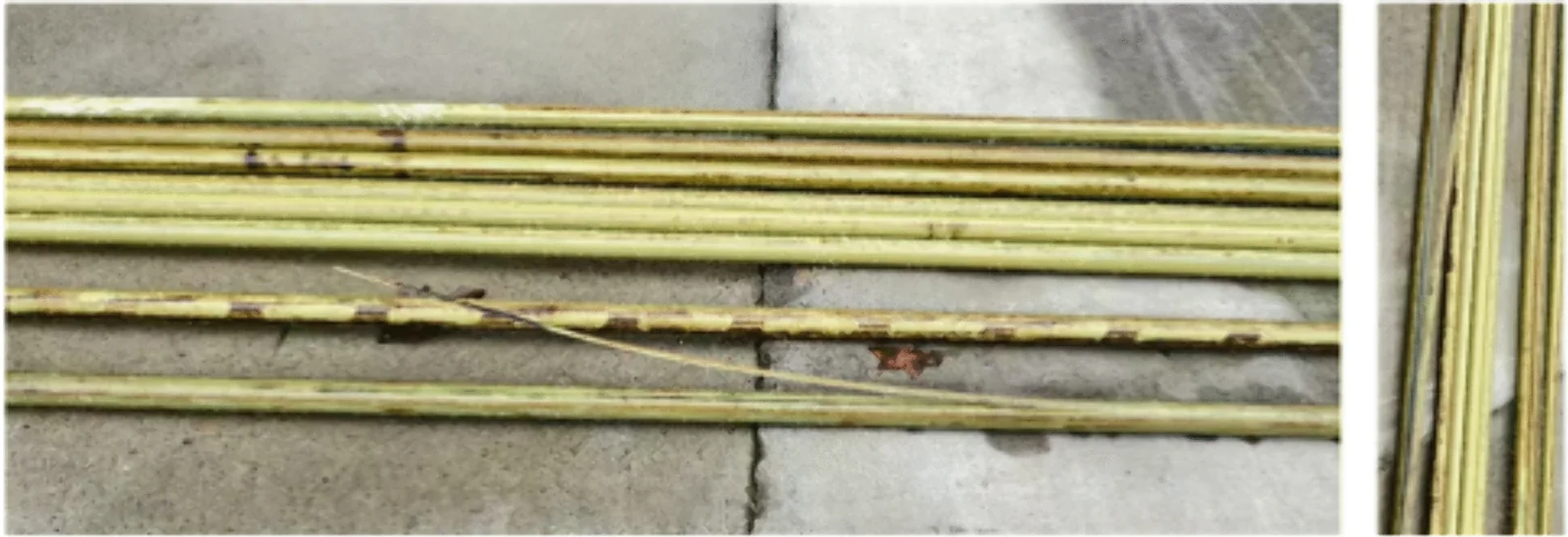
Field-observed longitudinal splitting of the PFRC during retrieval and drum winding.

### 2.2. Test materials and sample preparation

The investigated PFRC had a nominal diameter of 22 mm and was manufactured by pultrusion using SCF40-12K carbon fibers and OC WS3000 glass fibers. The carbon- and glass-fiber volume fractions were 29 ± 1% and 39 ± 1%, respectively, corresponding to a total fiber volume fraction of 68 ± 2%. The resin volume fraction was 32 ± 2%, and the measured bulk density of the rod was 1.9 g/cm^3^. The matrix consisted of an epoxy resin cured with an anhydride-based curing agent, with a degree of cure greater than 95%. All fibers were continuously aligned along the longitudinal axis of the rod. As shown in Fig 2, the radial architecture consisted of an outer glass-fiber layer, an intermediate carbon/glass hybrid transition region, and an inner carbon-fiber core. During pultrusion, the fibers were impregnated in an open resin bath maintained at 30 ± 3 °C and subsequently pulled through a 1.0 m-long heated die at a speed of 0.2 m/min. The inlet, intermediate, and outlet die temperatures were 147, 195, and 195 °C, respectively, and the pulling force was maintained below 2.5 t.

**Fig 2:**
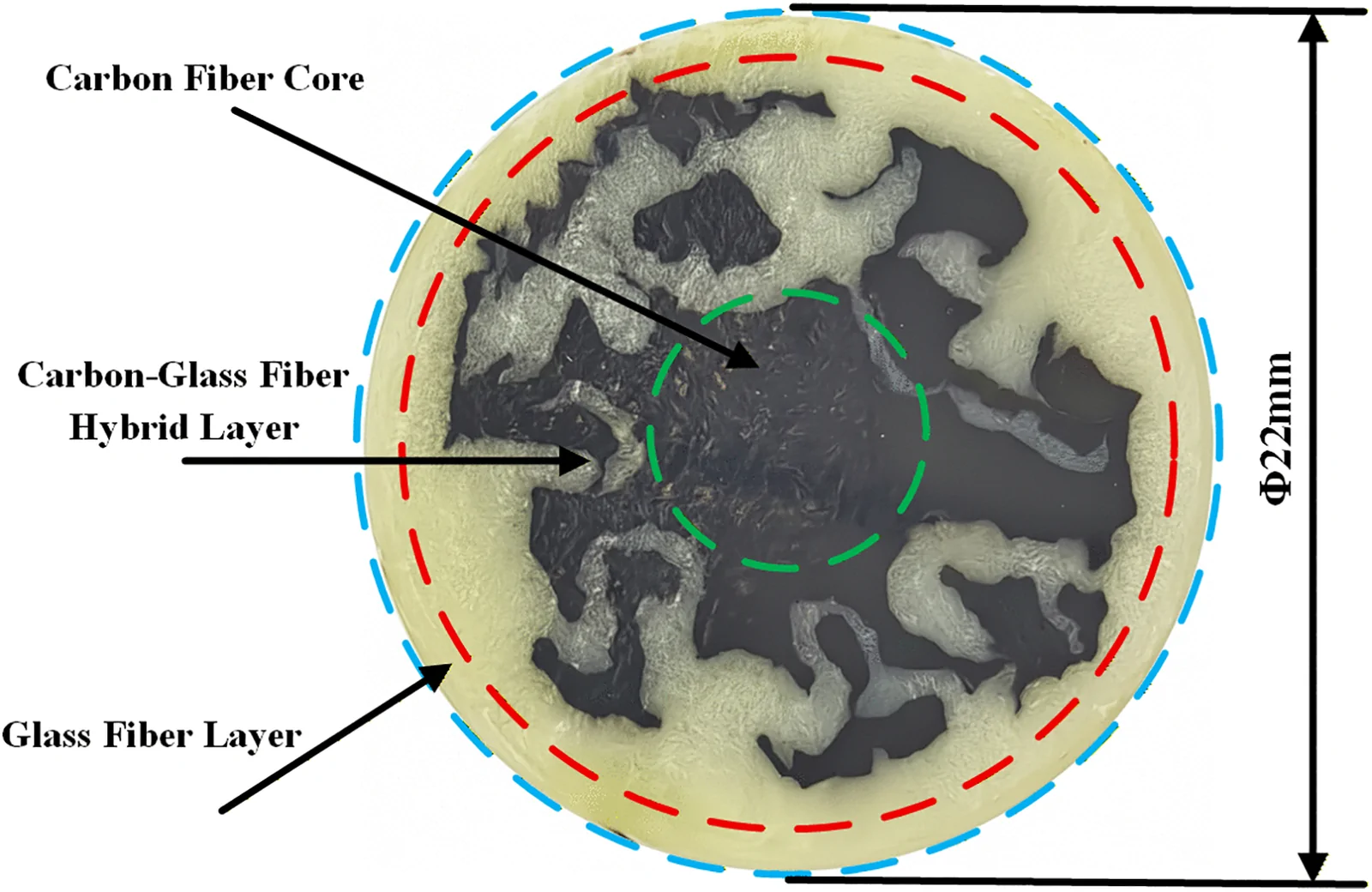
Schematic of the structural dimensions of PFRC.

The service-aged PFRC had operated in Well Yan 22−2 for 435 days. A 38 mm-diameter downhole pump was installed at a depth of approximately 3200 m, whereas the rod specimens examined in this study were obtained from a nearly vertical section at a depth of approximately 1000–1020 m. Because continuous downhole temperature and pressure profiles were unavailable, the local conditions at the sampling position were estimated from field operating data. Based on a surface temperature of 25 °C and a geothermal gradient of 2.3 °C/100 m, the temperature at the sampling depth of approximately 1000–1020 m was estimated to be 48.0–48.5 °C. The hydrostatic pressure, estimated using an average fluid density of 963.37 kg/m^3^, was approximately 9.45–9.64 MPa. Representative values of 48 °C and 9.55 MPa were therefore adopted. These values represent approximate service conditions rather than time-resolved exposure histories; accordingly, the individual effects of temperature and pressure cannot be quantitatively separated from those of fluid exposure and cyclic loading. This uncertainty does not affect the identification of the experimentally observed post-service degradation, but it limits the attribution of the observed damage to any individual environmental factor. The produced fluid had a water cut of 91.5%, a pH of 7.9, and a total dissolved solids content of 15,861 mg/L; CO_2_ and H_2_S were not detected. The pumping system operated with a stroke length of 6 m and a frequency of 1.5 cycles/min. Detailed environmental and operating parameters are summarized in Table 1.

**Table 1:** Environmental and operating conditions of the field-serviced PFRC.

Parameter	Value
Sampling depth	1000–1020 m
Surface reference temperature	25 °C
Geothermal gradient	2.3 °C/100 m
Estimated temperature at sampling position	48 °C
Estimated pressure at sampling position	9.55 MPa
Wellhead backpressure	0.39 MPa
Casing pressure	0.30 MPa
Average well-fluid density	963.37 kg/m³
Produced-fluid water cut	91.5%
pH	7.9
Total dissolved solids	15,861 mg/L
Ca^2+^	344 mg/L
Na^+^ + K^+^	5453 mg/L
Mg^2+^	93 mg/L
Cl^−^	8333 mg/L
HCO_3_^−^	1638 mg/L
CO_2_	Not detected
H_2_S	Not detected
Pump stroke	6 m
Pumping frequency	1.5 cycles/min
Maximum/minimum polished-rod loads	94.4/48.8kN
Estimated maximum/minimum loads at sampling position	74/32kN
Estimated accumulated loading cycles	9.40 × 10^5^

The axial loads at the sampling position were estimated from the rod-string load distribution. The upstroke and downstroke loads at the pump end were first calculated from the force-balance relations.


$$\begin{array}{c} F_{s}\left(0\right)=w_{ls}+f_{cp}+f_{tl}+f_{hu}+f_{ll}-p_{p} \end{array}$$ (1)

$$\begin{array}{c} F_{x}\left(0\right)=-\left(f_{v}+f_{cp}+f_{hd}\right) \end{array}$$ (2)

where *F*_*s*_(0) and *F*_*x*_(0) are the axial loads acting on the pump plunger at the rod-string bottom during the upstroke and downstroke, respectively (N), with tensile load taken as positive and compressive load as negative; *w*_*ls*_ is the liquid-column load acting on the plunger (N); *f*_*cp*_ is the friction force between the pump plunger and barrel (N); *f*_*tl*_ is the friction force between the well fluid and tubing (N); *f*_*hu*_ and *f*_*hd*_ are the loads associated with wellhead backpressure during the upstroke and downstroke, respectively (N); *f*_*ll*_ is the liquid-column inertial load (N); *p*_*p*_ is the load exerted by the gas in the pump on the plunger (N); and *f*_*v*_ is the resistance caused by fluid flow through the traveling-valve port (N).

Using the field operating parameters, the calculated upstroke and downstroke pump-end loads were approximately 29.12 and −4.96kN, respectively. Because the 1000–1020 m sampling interval was located in a nearly vertical section and the axial-load distribution along this section was approximated as linear with depth, the local maximum and minimum loads were estimated by linear interpolation between the polished-rod loads (94.4/48.8kN) and the pump-end loads. At a representative depth of 1000 m,


$$\begin{array}{c} F_{max}=94.4+\left(29.12-94.4\right)\frac{1000}{3200}\approx 74.0\text{kN} \end{array}$$ (3)

$$\begin{array}{c} F_{min}=48.8+\left(-4.96-48.8\right)\frac{1000}{3200}\approx 32.0\text{kN} \end{array}$$ (4)

The variation over the 1000–1020 m sampling interval was small; therefore, 74 and 32kN were adopted as representative maximum and minimum axial loads.

The accumulated number of loading cycles was estimated from the nominal pumping frequency and the total service duration as


$$\begin{array}{c} N=f_{p}\times 60\times 24\times t \end{array}$$ (5)

where *N* is the accumulated number of loading cycles, *f*_*p*_ is the pumping frequency (cycles/min), and *t* is the service duration (days). Assuming continuous operation at the nominal pumping frequency of 1.5 cycles/min throughout the 435-day service period,


$$\begin{array}{c} N=1.5\times 60\times 24\times 435=9.396\times 10^{\text{5}} \end{array}$$ (6)

The pristine and service-aged PFRC rods were manufactured in the same production batch using the same constituent materials, nominal fiber and resin contents, pultrusion process, and curing conditions. The service-aged specimens were consecutively sectioned from the same continuous rod segment retrieved from approximately 1000–1020 m depth. The pristine specimens were likewise consecutively sectioned from a single parent rod.

### 2.3. Mechanical property testing

#### 2.3.1 Tensile test.

As shown in Fig 3, tensile tests were conducted in accordance with GB/T 13096−2008 using specialized grips designed for PFRC specimens. The exposed length of each specimen was 145 mm. Tests were performed on a WAW-1000F universal testing machine under displacement control at a constant loading rate of 2 mm/min. The load–displacement response was continuously recorded until failure. Five specimens were tested for each condition.

**Fig 3:**
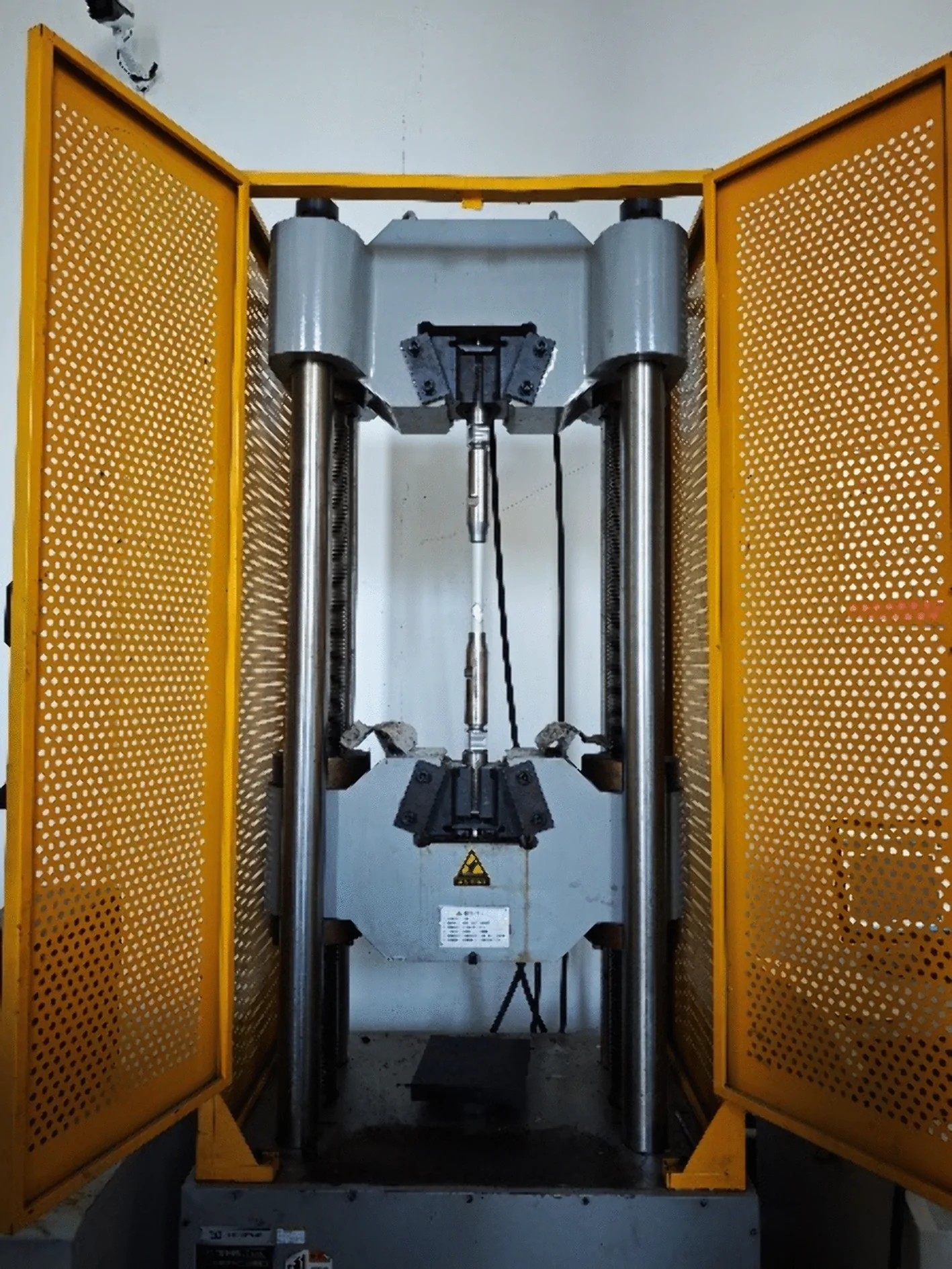
Experimental setup for tensile testing.


$$\begin{array}{c} \sigma_{\text{t}}=\frac{P_{\text{t}}\times 4}{\pi \times D^{\text{2}}} \end{array}$$ (7)

where *σ*_t_ is the tensile strength (MPa), *P*_t_ is the maximum tensile load (N), and *D* is the specimen diameter (mm).

#### 2.3.2 Compression test.

As shown in Fig 4, compression tests were conducted in accordance with GB/T 1448–2005. Cylindrical specimens with a diameter of 22 mm and a height of 25 mm were tested using a CMT5305 universal testing machine. Each specimen was continuously loaded until failure. Five specimens were tested for each condition.

**Fig 4:**
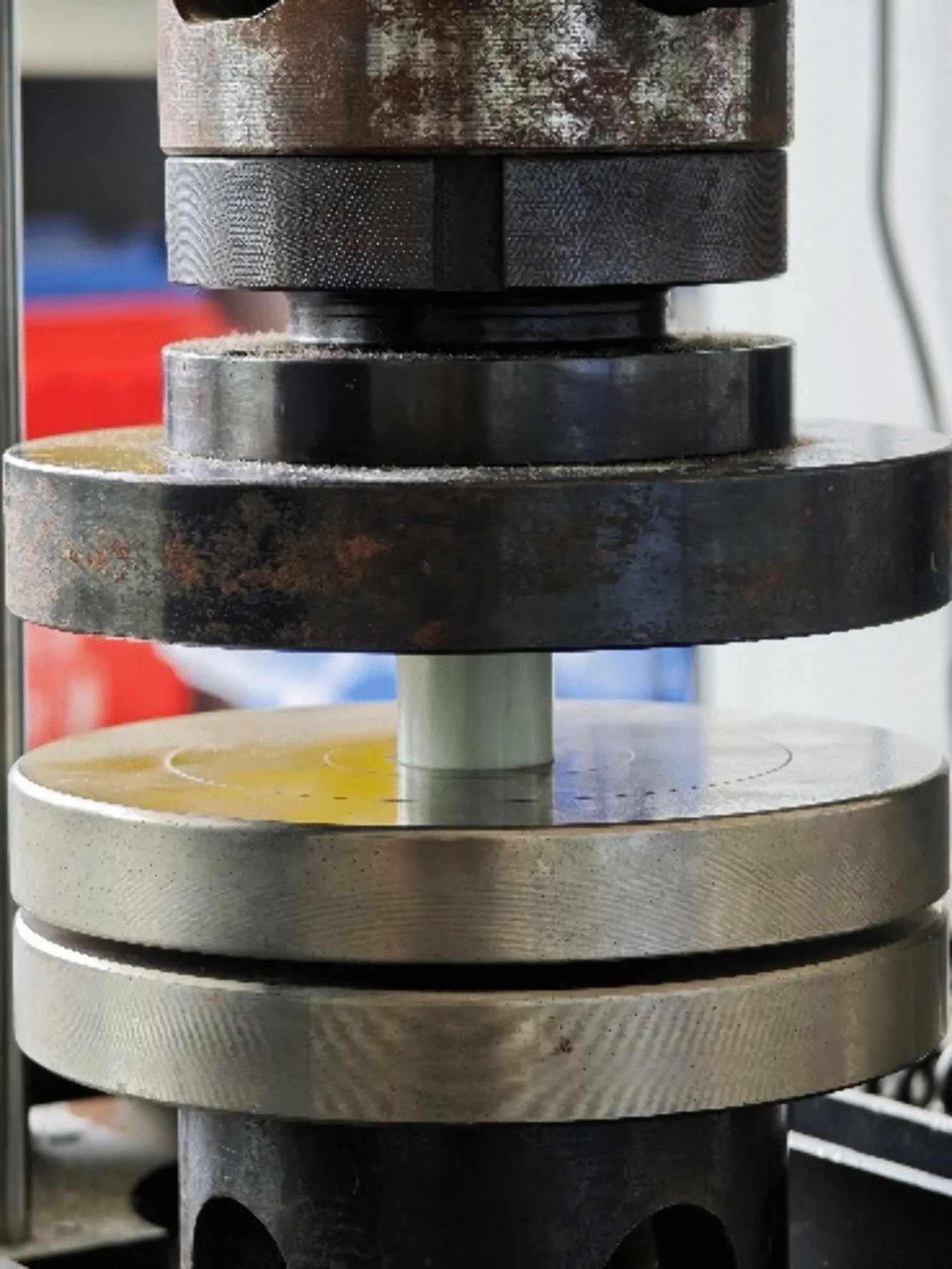
Experimental setup for compression testing.


$$\begin{array}{c} \sigma_{\text{c}}=\frac{P_{\text{c}}\times 4}{\pi \times D^{\text{2}}} \end{array}$$ (8)

where *σ*_c_ is the compressive strength (MPa), *P*_c_ is the maximum compressive load (N).

#### 2.3.3 Three-point bending test.

As shown in Fig 5, three-point bending tests were conducted in accordance with GB/T 13096−2008. Specimens 440 mm in length were placed on two supports with a span of 352 mm and tested using a CMT5305 universal testing machine. The load was applied at midspan under displacement control at a constant crosshead speed of 6 mm/min. Loading was terminated when either the fibers on the lower surface fractured or the midspan deflection reached 1.5 times the specimen height. Five specimens were tested for each condition.

**Fig 5:**
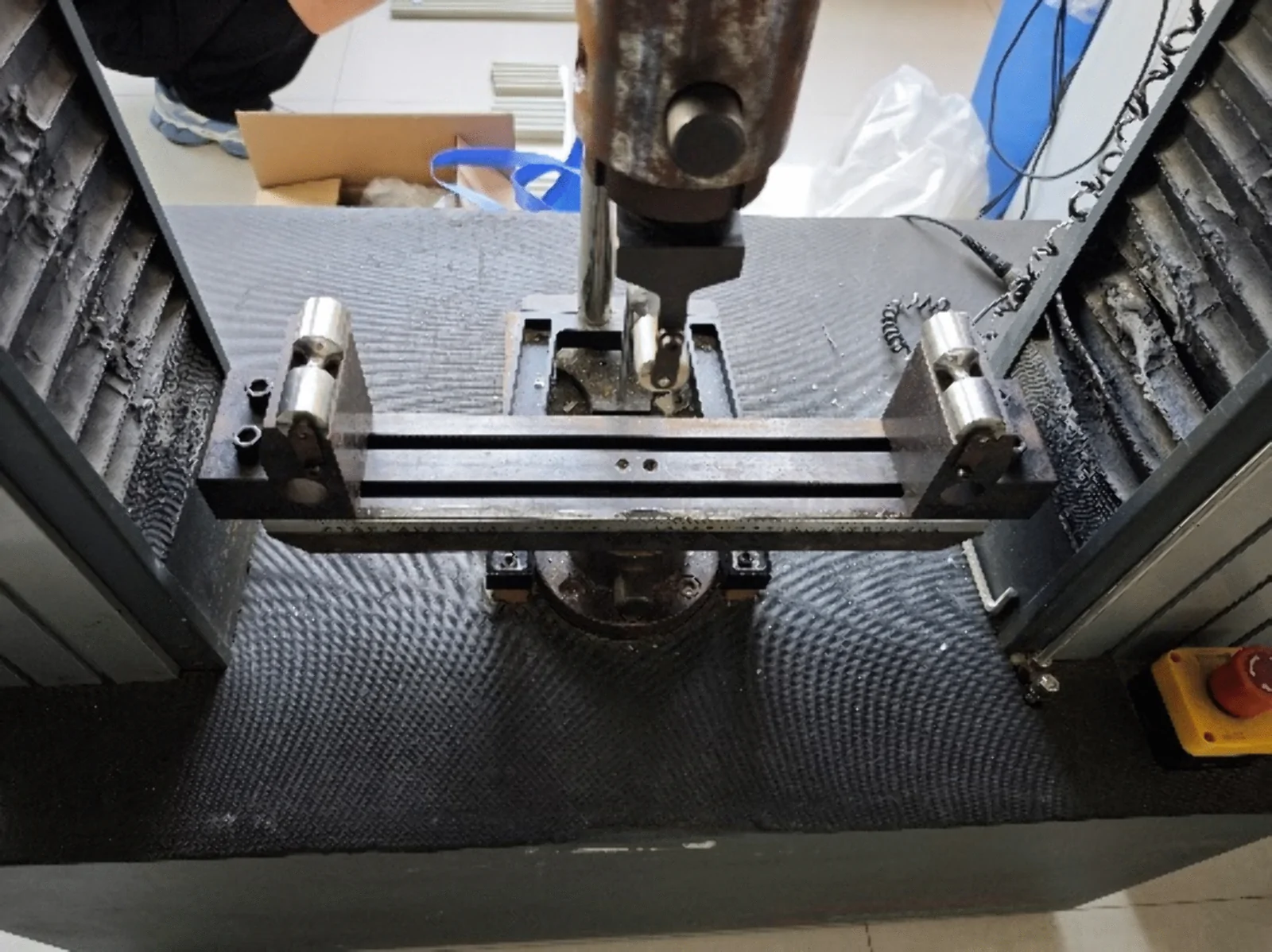
Experimental setup for three-point bending testing.


$$\begin{array}{c} \sigma_{f}=\frac{8\times P_{f}\times S}{\pi \times D^{\text{3}}} \end{array}$$ (9)

where *σ*_*f*_ is the flexural strength (MPa), *P*_*f*_ is the maximum load recorded during the three-point bending test (N), *S* is the support span (mm).

#### 2.3.4 Apparent horizontal shear test.

As shown in Fig 6, apparent horizontal shear tests were conducted in accordance with GB/T 13096−2008 using a short-beam three-point loading configuration. Specimens 200 mm in length were placed on two supports with a span of 90 mm and tested using a CMT5305 universal testing machine. A transverse load was applied through the loading head at a constant crosshead speed of 1.3 mm/min. The duration of each test was maintained within 20–200 s. Five specimens were tested for each condition.

**Fig 6:**
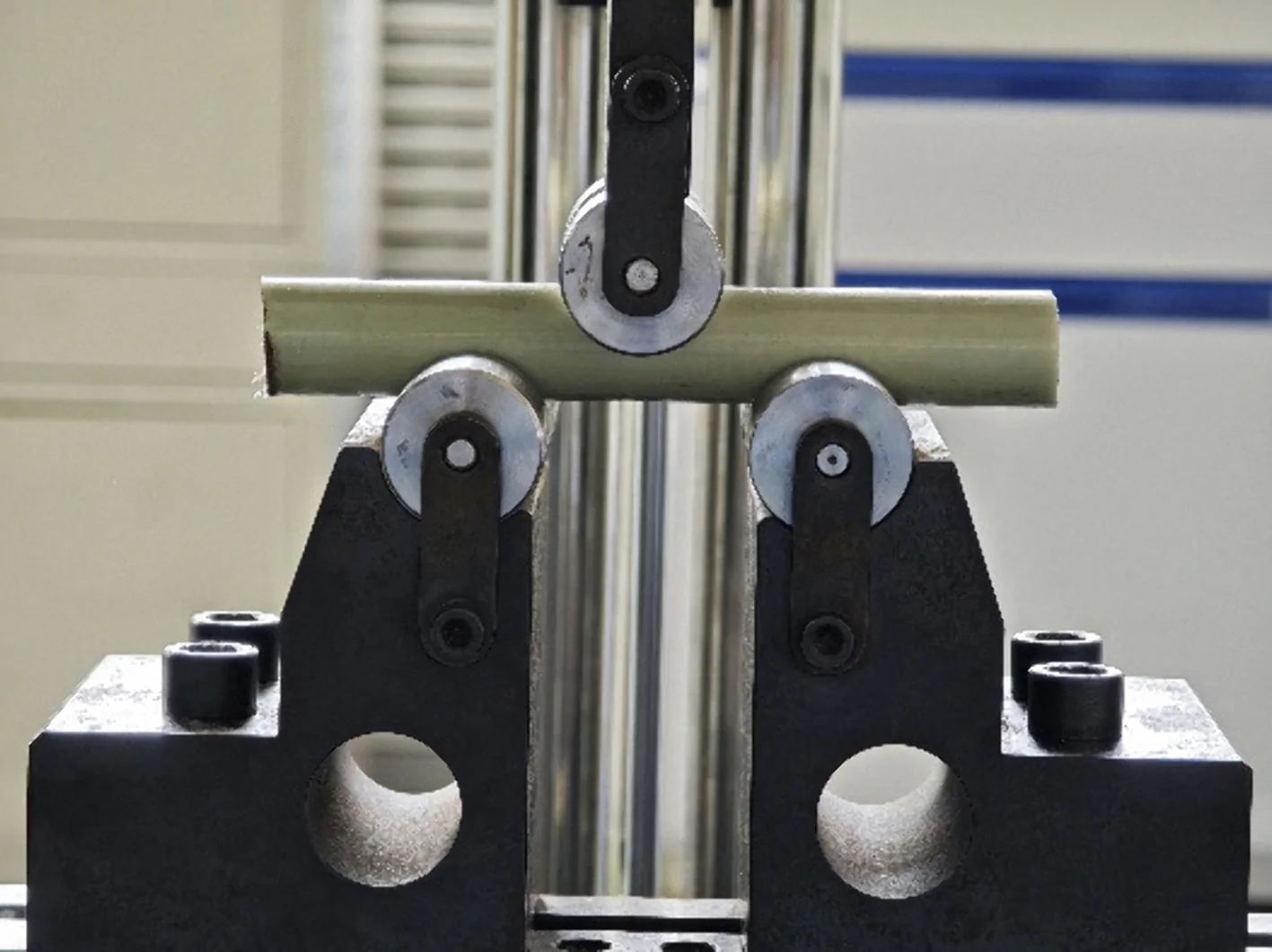
Experimental setup for apparent horizontal shear testing.


$$\begin{array}{c} \tau_{\text{s}}=\frac{8\times P_{\text{s}}}{3\times \pi \times D^{\text{2}}} \end{array}$$ (10)

where *τ*_s_ is the apparent short-beam shear strength (MPa), *P*_s_ is the maximum applied load (N). Because the circular hybrid rod is subjected to a complex stress state under the short-beam loading configuration, *τ*_s_ is treated as an apparent strength parameter rather than a direct measure of interfacial shear strength.

### 2.4. Dynamic mechanical analysis

Dynamic mechanical analysis (DMA) was performed to compare the thermomechanical behavior of the pristine and service-aged PFRC after 435 days of field service. Tests were conducted in accordance with ASTM D7028-07(2024). As shown in Fig 7, rectangular specimens measuring 17.5 mm × 7.5 mm × 2.2 mm were sectioned from the carbon-fiber core of the rods. DMA measurements were conducted in single-cantilever mode at an oscillation amplitude of 10.0 μm, a frequency of 1.0 Hz, and a heating rate of 5 °C/min. The storage modulus and tan *δ* were recorded as functions of temperature, and the glass-transition temperature (*T*_g_) was determined from the DMA response.

**Fig 7:**
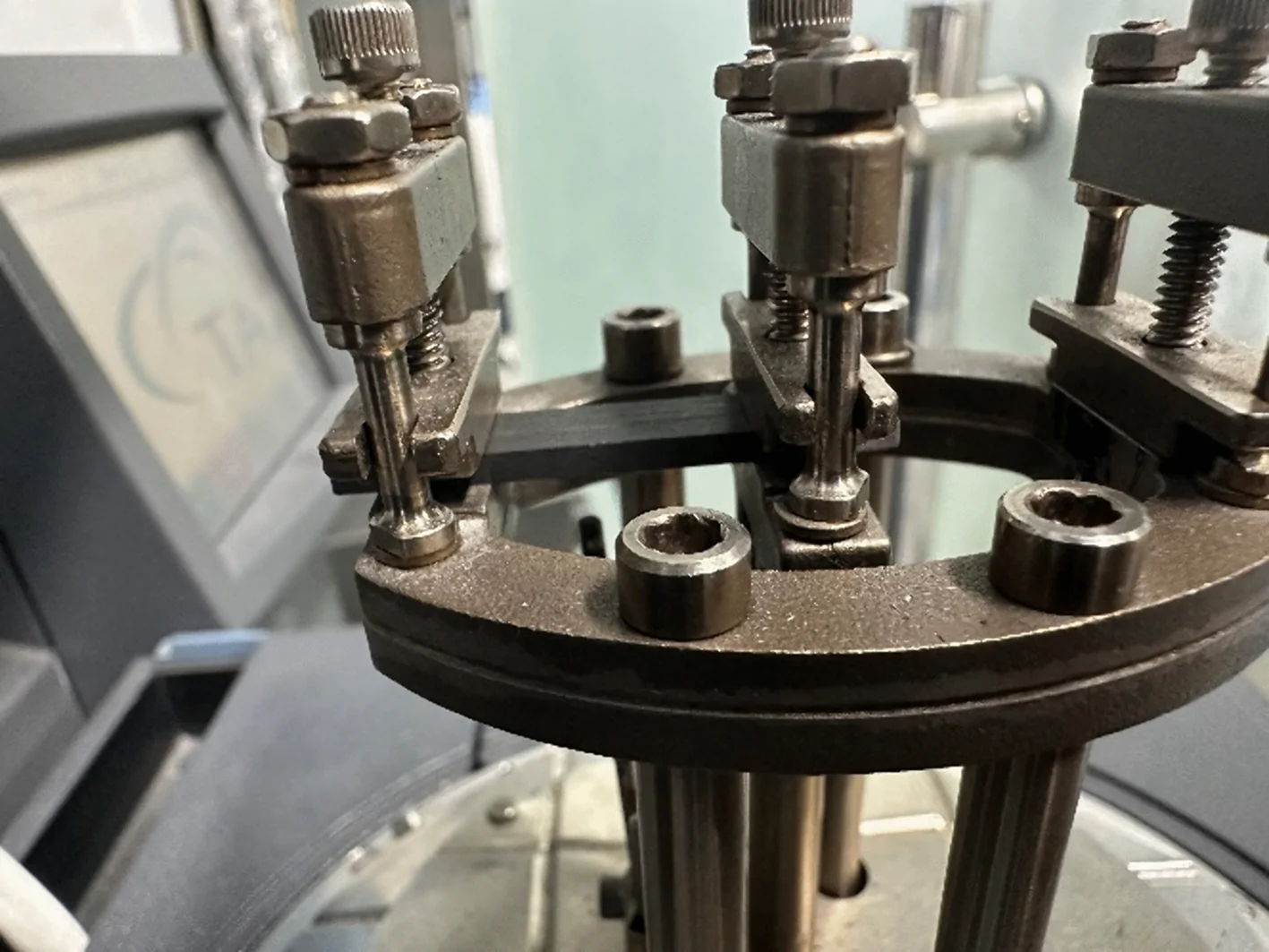
Experimental setup for DMA testing.

### 2.5. Scanning electron microscopy

The fracture morphologies of pristine and service-aged PFRC specimens were examined after mechanical testing using a TESCAN MIRA field-emission scanning electron microscope (SEM). Both transverse fracture surfaces and longitudinal sections were examined, with particular attention to fiber distribution, matrix fracture, fiber pull-out, and interfacial debonding within different structural regions. SEM specimens were prepared as cylindrical blocks approximately 22 mm in diameter and 20 mm in height. Before observation, the specimens were vacuum-coated with gold to improve electrical conductivity. In addition, quantitative image analysis was performed using ImageJ to supplement the qualitative fractographic observations. Quantitative SEM analysis was performed using ImageJ based on three representative fields for each failure mode and condition. ROIs were defined around clearly identifiable target features, and all features satisfying the same measurement criteria within each ROI were measured; regions with ambiguous boundaries or imaging artifacts were excluded. Because feature density varied with fracture morphology, the numbers of ROIs and individual measurements were not fixed.

### 2.6. Moisture desorption test method

To compare the removable moisture retained in pristine and service-aged PFRC specimens, moisture-desorption tests were conducted by monitoring mass loss during controlled drying. The equilibrium mass-loss ratio was used as a comparative indicator of removable moisture retention rather than as a direct measure of moisture uptake during field service.

Specimens representing the whole rod, the shell layer, and the core layer were prepared from both new and service-exposed rods. Each specimen was weighed to establish its initial mass. Subsequently, the specimens were categorized and placed in an electric thermostatic drying oven maintained at (60 ± 2 °C). The specimens were periodically removed for weighing at specific intervals: 1 day, 3 days, and 7 days, followed by weekly measurements thereafter. The experiment concluded once the mass of the specimens reached a stable state. Upon completion of the testing, the dehydration rates for the three types of specimens were calculated, mass loss curves were plotted, and the experimental data were subjected to error analysis and compilation.


$$\begin{array}{c} M_{\text{t}}=\frac{m_{\text{0}}-m_{\text{t}}}{m_{\text{0}}}\times 100 \end{array}$$ (11)

where $M_{\text{t}}$ represents the dehydration rate of PFRC specimen, %; $m_{\text{t}}$ denotes the mass of PFRC specimen after drying in a thermostatic oven for a specified duration, g; $m_{\text{0}}$ is the initial mass of PFRC specimen, g.

### 2.7. Statistical analysis

Mechanical-property data are reported as mean ± standard deviation (SD), with five specimens tested for each condition. Differences between the pristine and service-aged groups were evaluated using two-tailed independent-samples Welch’s t-tests at *α* = 0.05. Tensile, compressive, flexural, and apparent short-beam shear strengths were treated as four prespecified mechanical responses. Because multiple hypothesis tests were performed on the same set of mechanical outcomes, adjustment was applied to control the family-wise Type I error rate. The Holm procedure was selected because it provides strong family-wise error control while being less conservative than the classical Bonferroni correction. Holm-adjusted p-values were therefore calculated in addition to the individual Welch’s t-test p-values.

Strength distributions were further evaluated using a two-parameter Weibull model [30]. For the median-rank least-squares (LS) analysis, the cumulative failure probability assigned to the *i*th strength value in ascending order was first estimated from


$$\begin{array}{c} F_{i}=\frac{i-0.3}{n+0.4} \end{array}$$ (12)

where *n* denotes the number of specimens in the corresponding group. The median-rank LS approach was selected as the primary fitting method because it provides a direct Weibull probability-plot representation of the ordered strength data and allows the shape and scale parameters to be obtained transparently from the linearized Weibull relation. Given the small sample size, MLE was additionally applied to assess the sensitivity of the fitted parameters to the estimation method.

The ranked strength data were then described by the two-parameter Weibull cumulative distribution


$$\begin{array}{c} F\left(\sigma \right)=1-e^{-{\left(\frac{\sigma }{\sigma_{\text{0}}}\right)}^{m}} \end{array}$$ (13)

with *m* representing the Weibull shape parameter and *σ*_0_ the characteristic strength at a cumulative failure probability of 63.2%. Higher *m* values correspond to lower strength dispersion. Taking the double logarithm of Eq (13) gives


$$\begin{array}{c} ln\left(ln\left(\frac{\text{1}}{1-F}\right)\right)=mln\sigma -mln\sigma_{\text{0}} \end{array}$$ (14)

which permits *m* and *σ*_0_ to be obtained from a linear regression of the transformed data. The regression slope gives *m*. With *b* denoting the fitted intercept, the scale parameter follows from


$$\begin{array}{c} \sigma_{\text{0}}={\text{e}}^{\left(-\frac{b}{m}\right)} \end{array}$$ (15)

For comparison with the median-rank LS estimates, the same strength datasets were also fitted by maximum likelihood estimation (MLE). Under the same two-parameter Weibull model, the probability density function takes the form


$$\begin{array}{c} f\left(\sigma ;m,\sigma_{\text{0}}\right)=\frac{m}{\sigma_{\text{0}}}{\left(\frac{\sigma }{\sigma_{\text{0}}}\right)}^{m-1}e^{-{\left(\frac{\sigma }{\sigma_{\text{0}}}\right)}^{m}} \end{array}$$ (16)

For *n* measured strength values, the corresponding log-likelihood function becomes


$$\begin{array}{c} \ell \left(m,\sigma_{\text{0}}\right)=nlnm-nmln\sigma_{\text{0}}+\left(m-1\right)\sum_{i=1}^{n}ln\sigma_{i}-{\sum_{i=1}^{n}\left(\frac{\sigma_{i}}{\sigma_{\text{0}}}\right)}^{m} \end{array}$$ (17)

The MLE values of *m* and *σ*_0_ were obtained by numerical maximization of Eq (17), with the location parameter fixed at zero. The resulting estimates were compared with the LS values for each mechanical property.

Uncertainty in the LS-derived Weibull parameters was assessed by parametric bootstrap analysis. For each specimen condition, 10,000 synthetic datasets containing five observations were generated from the fitted Weibull distribution and analyzed using the same median-rank LS procedure. The 2.5th and 97.5th percentiles of the resulting parameter distributions were taken as the 95% bootstrap confidence limits. Given the five specimens available for each condition, the Weibull results were used to characterize strength-distribution trends rather than to provide design-level reliability parameters.

Descriptive statistics, confidence intervals, and Welch’s t-tests were calculated using Microsoft Excel 2021. Weibull fitting and bootstrap analysis were performed using author-generated code in MATLAB R2025b. The analysis code and underlying data are available at 10.5281/zenodo.21915719.

## 3. Results and discussion

### 3.1. Mechanical properties

#### 3.1.1. Tensile properties test results.

As illustrated in Fig 8(a, b), the tensile load–displacement curves of both the pristine and service-aged PFRC specimens exhibit predominantly linear elastic behavior during the initial loading stage. As the load approaches the ultimate capacity, minor load drops and serrated fluctuations appear, which are associated with the progressive stochastic fracture of individual fiber bundles before the final macroscopic failure of the composite rod. The tensile strength results and statistical comparison are summarized in Table 2. The mean tensile strength decreased from 1283.27 ± 62.92 MPa for the pristine PFRC to 1154.68 ± 83.57 MPa for the service-aged PFRC, corresponding to a reduction of 10.02% and a strength retention of 89.98%. The Welch’s t-test gave a mean difference of 128.58 MPa (95% confidence interval: 19.25–237.92 MPa), with an unadjusted p-value of 0.029. After Holm adjustment, the p-value increased to 0.057 and no longer met the 0.05 significance criterion. The standard deviation increased from 62.92 to 83.57 MPa after service, indicating greater scatter in tensile strength among the service-aged specimens. For the present pumping system, the maximum polished-rod load was 94.4kN (approximately 9.6tf). Based on the 22 mm rod diameter, the mean tensile strength of 1154.68 MPa after 435 days of service corresponds to an axial failure load of approximately 439kN. Thus, although the tensile strength decreased by 10.02% after service, the residual static tensile capacity remained well above the maximum axial load encountered in the present well.

**Table 2:** Tensile strength results and statistical comparison of pristine and service-aged PFRC.

Specimen state	Mean ± SD (MPa)	Mean difference (MPa)	95% CI of mean difference (MPa)	t	df	Unadjusted p-value	Holm-adjusted p-value
pristine	1283.27 ± 62.92	128.584	19.25–237.92	2.748	7.432	0.029	0.057
service-aged	1154.68 ± 83.57						

**Fig 8:**
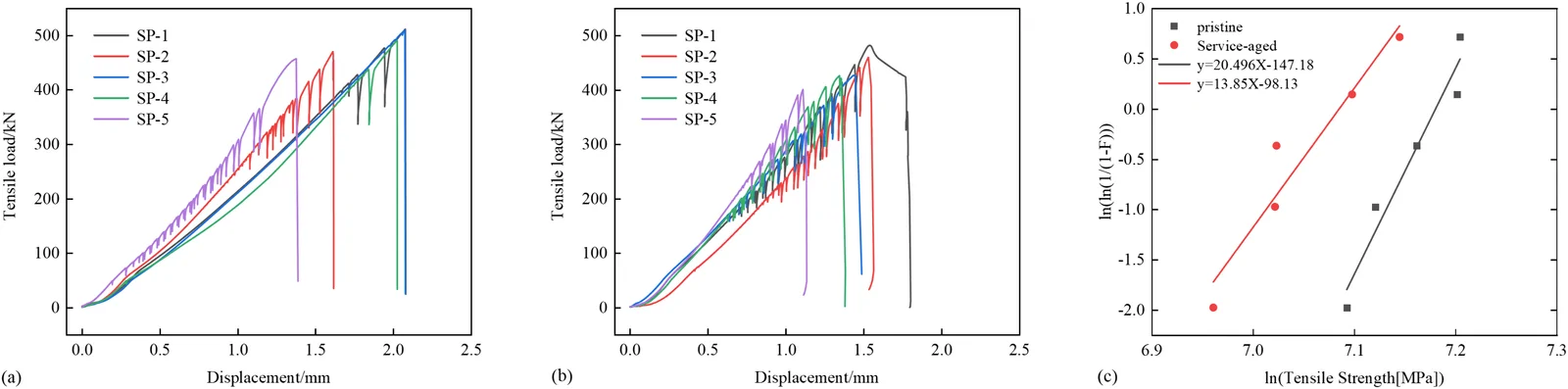
Tensile response and Weibull analysis of pristine and service-aged PFRC: (a) load-displacement curves of pristine PFRC; (b) load-displacement curves of service-aged PFRC; (c) Weibull regression plots.

The Weibull results are summarized in Fig 8(c) and Table 3. The median-rank LS fit gave shape parameters of 20.49 for the pristine PFRC and 13.85 for the service-aged PFRC. The corresponding 95% bootstrap confidence intervals, 8.24–52.23 and 5.54–35.55, were broad and strongly overlapping, indicating considerable uncertainty in the absolute *m* values. The LS scale parameter decreased from 1313.11 to 1193.87 MPa. MLE shifted the shape-parameter estimates upward to 27.75 and 16.66, respectively, but produced very similar scale parameters of 1309.70 and 1190.47 MPa. Thus, the estimate of *σ*_0_ was relatively insensitive to the fitting method, whereas *m* showed greater method dependence. Despite this difference in absolute *m*, both fits placed the service-aged specimens at lower characteristic strength and greater tensile-strength dispersion, consistent with the reduction in mean strength and the increase in experimental scatter. Given the limited sample size, the change in *m* is interpreted as a distributional trend rather than as a precise estimate of the population-level change.

**Table 3:** Weibull parameters obtained using median-rank least-squares fitting and maximum likelihood estimation for tensile strength.

Specimen state	Experimental mean (MPa)	LS *m*	95% bootstrap CI of LS *m*	MLE *m*	LS *σ*_0_ (MPa)	95% bootstrap CI of LS *σ*_0_ (MPa)	MLE *σ*_0_ (MPa)	LS *R*^2^
Pristine	1283.27	20.49	8.24–52.23	27.75	1313.11	1246.57–1368.8	1309.7	0.948
Service-aged	1154.68	13.85	5.54–35.55	16.66	1193.87	1106.63–1269.91	1190.47	0.924

The tensile failure morphology was consistent with these statistical trends. As shown in Fig 9(a), the pristine PFRC exhibited relatively localized fracture dominated by concentrated fiber breakage, consistent with its comparatively narrow strength distribution. In contrast, the service-aged specimen showed more extensive longitudinal splitting and less coordinated fiber-bundle failure (Fig 9(b)). These features are consistent with weakened interfacial stress transfer and the greater tensile-strength dispersion observed after service.

**Fig 9:**
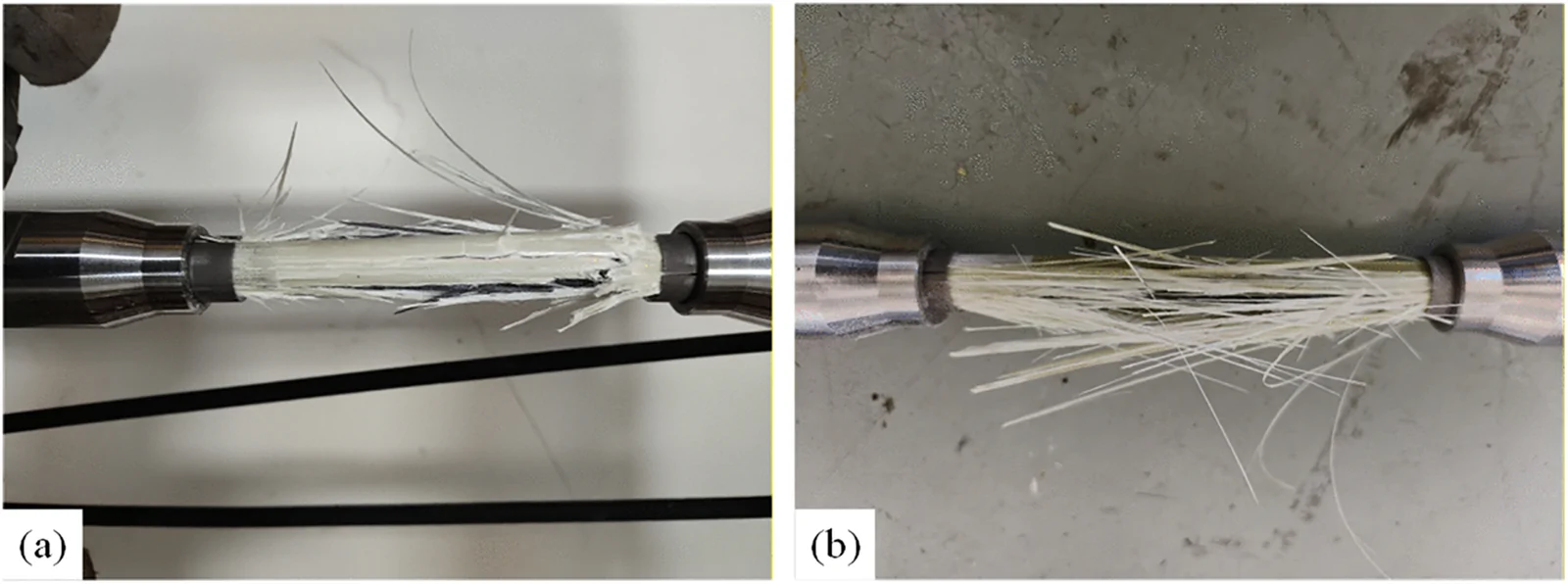
Macroscopic tensile failure morphologies of pristine and service-aged PFRC: (a) Pristine PFRC; (b) Service-aged PFRC.

#### 3.1.2. Compressive performance test results.

As shown in Fig 10(a, b), the compressive load–displacement curves of the pristine PFRC specimens exhibit a highly consistent response, with the five curves closely overlapping throughout the loading process. In contrast, the service-aged specimens show substantially greater scatter in both the ultimate compressive load and the corresponding failure displacement, although no obvious reduction in the initial compressive stiffness is observed. The compressive strength results and statistical comparison are summarized in Table 4. The mean compressive strength changed from 554.70 ± 13.41 MPa for the pristine PFRC to 542.21 ± 102.38 MPa for the service-aged PFRC, corresponding to a numerical reduction of approximately 2.25%. A two-tailed independent-samples Welch’s t-test showed that the difference in mean compressive strength was not statistically significant, with a mean difference of 12.49 MPa (95% confidence interval: −114.07 to 139.04 MPa, p = 0.800). This conclusion remained unchanged after Holm correction across the four prespecified mechanical-property comparisons (adjusted p = 0.800). However, the standard deviation increased markedly from 13.41 to 102.38 MPa after field service, indicating a pronounced increase in compressive-strength dispersion.

**Table 4:** Compressive strength results and statistical comparison of pristine and service-aged PFRC.

Specimen state	Mean ± SD (MPa)	Mean difference (MPa)	95% CI of mean difference (MPa)	t	df	Unadjusted p-value	Holm-adjusted p-value
pristine	554.70 ± 13.41	12.488	−114.07–139.04	0.27	4.137	0.800	0.800
service-aged	542.21 ± 102.38						

**Fig 10:**
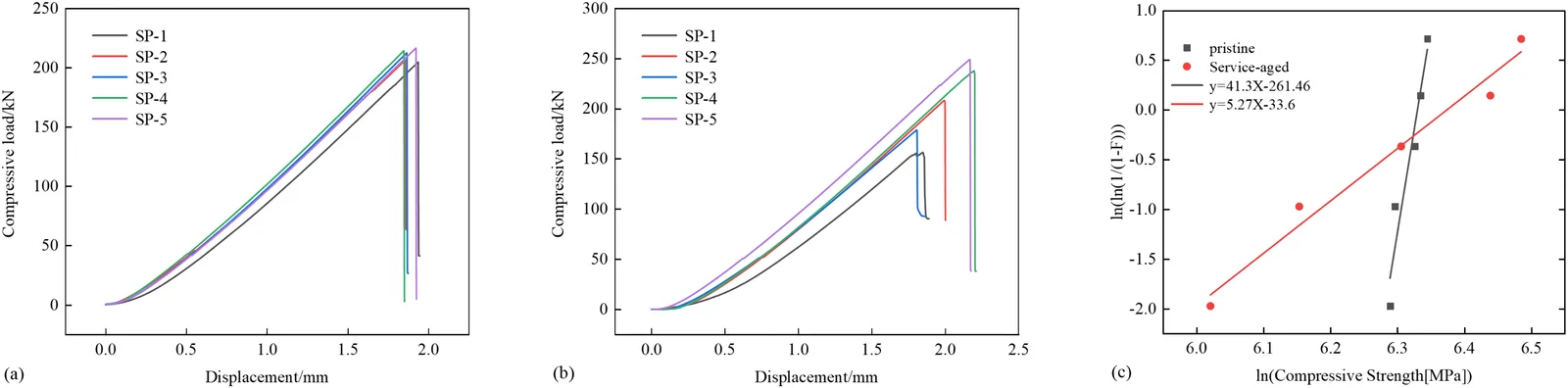
Compressive response and Weibull analysis of pristine and service-aged PFRC: (a) load–displacement curves of pristine specimens; (b) load–displacement curves of service-aged specimens; (c) Weibull regression plots.

The Weibull results are presented in Fig 10(c) and Table 5. The median-rank LS fit gave *m* = 41.31 for the pristine PFRC and *m* = 5.27 for the service-aged PFRC, with 95% bootstrap confidence intervals of 16.46–102.01 and 2.11–13.12, respectively. The corresponding scale parameter changed from 561.20 to 586.36 MPa. MLE produced higher absolute *m* values of 56.33 and 7.05, while the corresponding *σ*_0_ values were 560.41 and 580.95 MPa. Despite the difference in the absolute *m* estimates, both fitting methods showed a pronounced decrease in the shape parameter after service. This change agrees with the increase in the experimental coefficient of variation from 2.42% to 18.88% and indicates a substantial broadening of the compressive-strength distribution. The broad bootstrap intervals also show that the absolute values of *m* are not tightly constrained by the present sample size.

**Table 5:** Weibull parameters obtained using median-rank least-squares fitting and maximum likelihood estimation for compressive strength.

Specimen state	Experimental mean (MPa)	LS *m*	95% bootstrap CI of LS *m*	MLE *m*	LS *σ*_0_ (MPa)	95% bootstrap CI of LS *σ*_0_ (MPa)	MLE *σ*_0_ (MPa)	LS *R*^2^
Pristine	554.7	41.31	16.46–102.01	56.33	561.2	547.08–572.94	560.41	0.931
Service-aged	542.21	5.27	2.11–13.12	7.05	586.36	479.66–689.55	580.95	0.976

The increase in the Weibull scale parameter after field service does not imply an improvement in compressive strength. The parameter *σ*_0_ represents the characteristic strength at a cumulative failure probability of 63.2%, whereas the mean strength also depends on the Weibull shape parameter *m*. Based on the LS parameters, the fitted mean strengths were 553.68 MPa for the pristine PFRC and 540.01 MPa for the service-aged PFRC, close to the measured values of 554.70 and 542.21 MPa, respectively. The higher *σ*_0_ of the service-aged specimens therefore reflects the marked change in distribution shape: *m* decreased from 41.31 to 5.27, while the experimental coefficient of variation increased from 2.42% to 18.88%. MLE showed the same combination of a slightly higher scale parameter and a much lower shape parameter after service. These results indicate that the dominant change in compressive behavior was the substantial increase in strength variability rather than an increase in compressive strength.

The compressive failure morphology was also consistent with the increased strength dispersion observed after service. As shown in Fig 11(a, b), the pristine PFRC retained a relatively cohesive overall geometry despite longitudinal splitting, radial cracking, and localized interfacial damage. In contrast, the service-aged specimens exhibited penetrating longitudinal splitting, more extensive crushing, and severe radial cracking (Fig 11(c, d)). These morphological differences indicate a more heterogeneous compressive failure process after field service and are consistent with the substantially increased experimental scatter and lower Weibull shape-parameter estimate.

**Fig 11:**
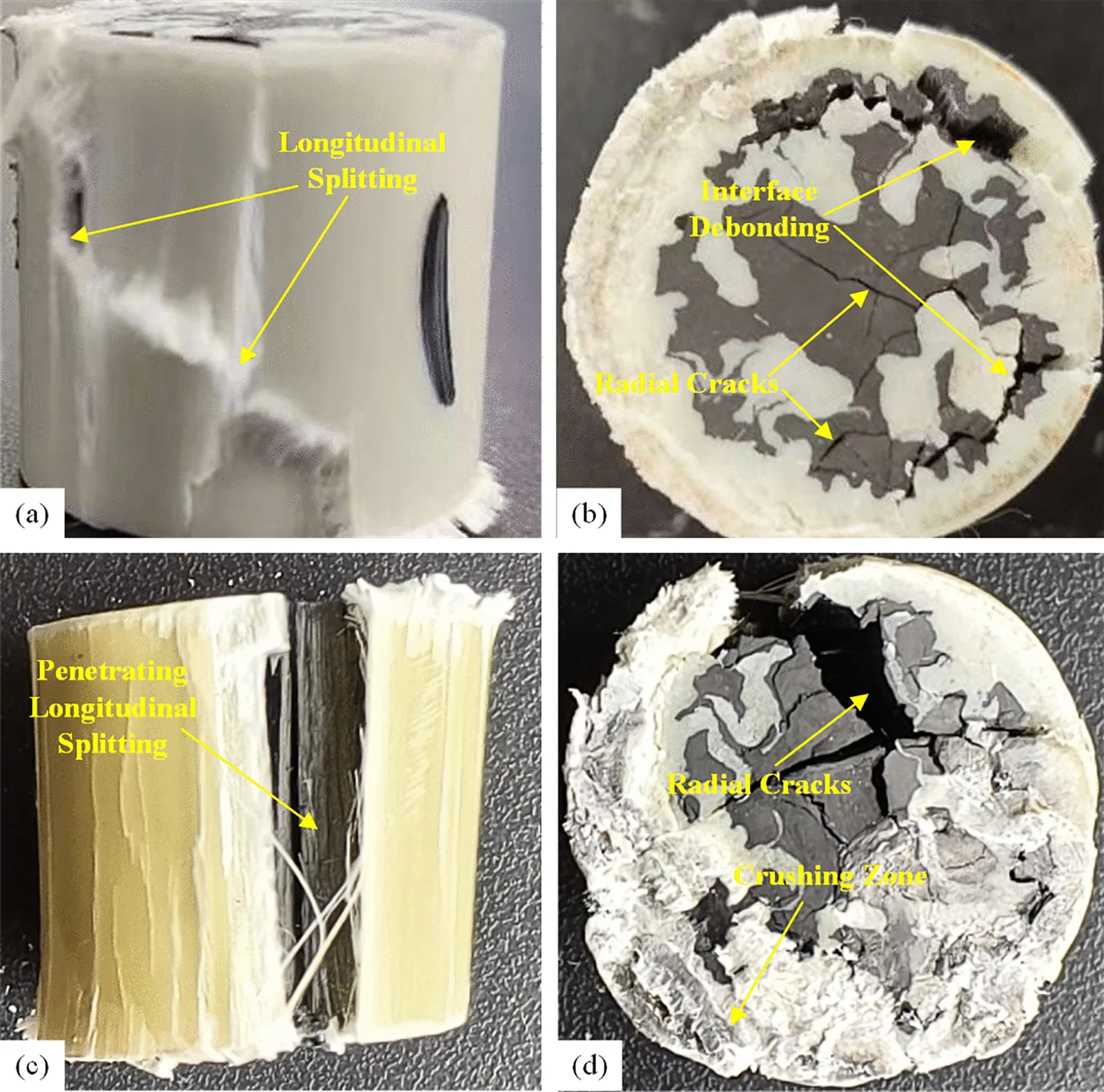
Macroscopic compressive failure morphologies of pristine and service-aged PFRC: (a) Pristine PFRC; (b) cross-section of pristine PFRC; (c) Service-aged PFRC; (d) cross-section of service-aged PFRC.

#### 3.1.3. Three-point bending performance test results.

As shown in Fig 12(a, b), the flexural load–displacement curves of both the pristine and service-aged PFRC specimens exhibit an approximately linear response during the initial loading stage, followed by a sudden load drop at failure, which is characteristic of brittle flexural fracture. The initial slopes of the curves remain relatively similar before and after service, whereas the ultimate flexural load and failure displacement decrease after 435 days of field service. The service-aged specimens also exhibit greater specimen-to-specimen variation than the pristine specimens. The flexural strength results and statistical comparison are summarized in Table 6. The mean flexural strength decreased from 1298.18 ± 34.30 MPa for the pristine PFRC to 1039.56 ± 43.53 MPa for the service-aged PFRC, corresponding to a reduction of approximately 19.92%. A two-tailed independent-samples Welch’s t-test confirmed that this reduction was statistically significant, with a mean difference of 258.62 MPa (95% confidence interval: 200.91–316.32 MPa, p < 0.001). The reduction remained statistically significant after Holm correction across the four prespecified mechanical-property comparisons (adjusted p < 0.001). In addition, the standard deviation increased from 34.30 to 43.53 MPa after service, indicating a moderate increase in the dispersion of flexural strength.

**Table 6:** Flexural strength results and statistical comparison of pristine and service-aged PFRC.

Specimen state	Mean ± SD (MPa)	Mean difference (MPa)	95% CI of mean difference (MPa)	t	df	Unadjusted p-value	Holm-adjusted p-value
pristine	1298.18 ± 34.3	258.616	200.91–316.32	10.434	7.585	<0.001	<0.001
service-aged	1039.56 ± 43.53						

**Fig 12:**
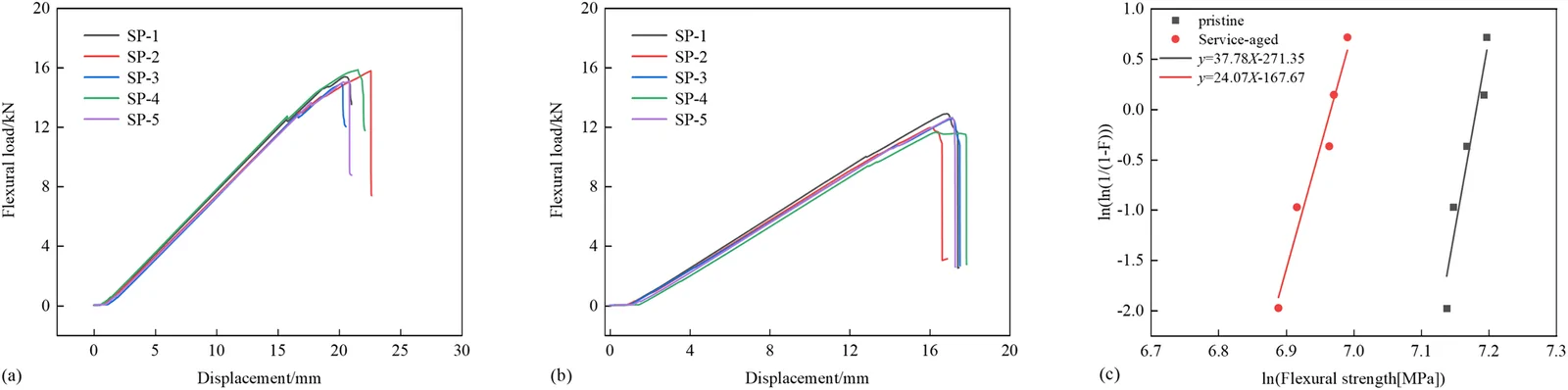
Flexural response and Weibull analysis of pristine and service-aged PFRC: (a) load–displacement curves of pristine specimens; (b) load–displacement curves of service-aged specimens; (c) Weibull regression plots.

The Weibull results are presented in Fig 12(c) and Table 7. The median-rank LS fit gave *m* = 37.78 for the pristine PFRC and *m* = 24.07 for the service-aged PFRC, with 95% bootstrap confidence intervals of 15.23–97.27 and 9.55–61.33, respectively. The corresponding scale parameter decreased from 1314.78 to 1060.23 MPa. MLE gave higher shape-parameter estimates of 49.14 and 33.61, but the scale parameters remained nearly unchanged relative to the LS results, at 1313.10 and 1057.61 MPa. The close agreement in *σ*_0_ indicates that the reduction in characteristic flexural strength is largely insensitive to the fitting method. In contrast, the absolute value of *m* varies more noticeably between LS and MLE, and the broad bootstrap intervals further indicate considerable uncertainty in the magnitude of the change in flexural-strength dispersion.

**Table 7:** Weibull parameters obtained using median-rank least-squares fitting and maximum likelihood estimation for flexural strength.

Specimen state	Experimental mean (MPa)	LS *m*	95% bootstrap CI of LS *m*	MLE *m*	LS *σ*_0_ (MPa)	95% bootstrap CI of LS *σ*_0_ (MPa)	MLE *σ*_0_ (MPa)	LS *R*^2^
Pristine	1298.18	37.78	15.23–97.27	49.14	1314.78	1278.02–1344.72	1313.1	0.927
Service-aged	1039.56	24.07	9.55–61.33	33.61	1060.23	1013.68–1097.8	1057.61	0.958

The flexural failure morphology was consistent with the observed mechanical degradation. As shown in Fig 13(a–c), the pristine PFRC exhibited relatively localized damage near the region of maximum bending moment, including a circumferential crack and a contained axial crack, while the cross-section retained substantial overall integrity. In contrast, the service-aged specimens exhibited more extensive longitudinal delamination, fiber fracture, and internal layering (Fig 13(d–f)). These features are consistent with weakened interlaminar constraint and more extensive interfacial damage after field service, which may have contributed to the reduction in flexural strength and the increased experimental scatter.

**Fig 13:**
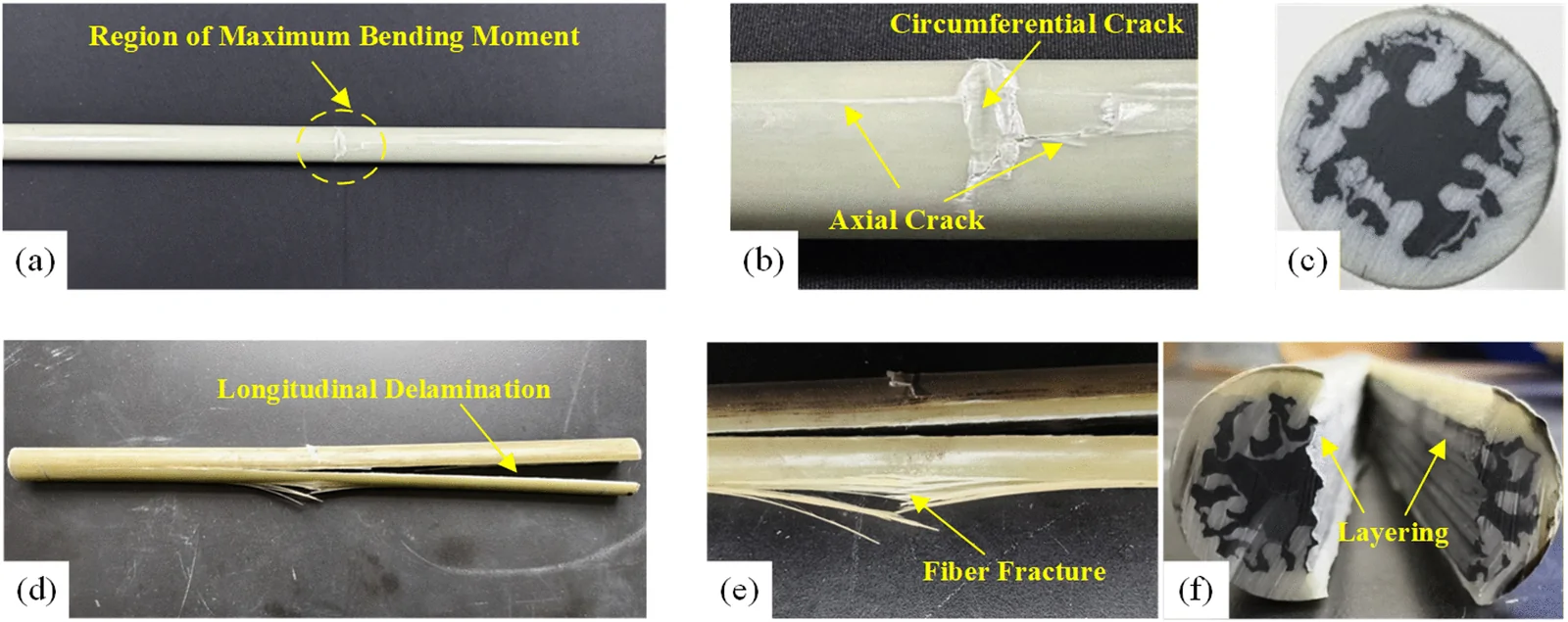
Macroscopic flexural failure morphologies of pristine and service-aged PFRC: (a) Pristine PFRC; (b) central failure region of the pristine specimen; (c) cross-section of the pristine specimen; (d) Service-aged PFRC; (e) central failure region of the service-aged specimen; (f) cross-section of the service-aged specimen.

#### 3.1.4 Apparent horizontal shear test results.

As shown in Fig 14(a, b), the load–displacement curves obtained from the apparent horizontal shear tests of both the pristine and service-aged PFRC specimens exhibit an approximately linear response during the initial loading stage, followed by a sudden load drop at failure. The sudden load drop indicates a brittle failure response under the short-beam loading configuration, in which interlaminar and interfacial damage contributed to the overall failure behavior. Although the overall shapes of the curves remain similar before and after field service, the service-aged specimens exhibit substantially lower ultimate loads. The apparent short-beam shear strength results and statistical comparison are summarized in Table 8. The mean apparent short-beam shear strength decreased from 70.76 ± 1.77 MPa for the pristine PFRC to 55.21 ± 1.71 MPa for the service-aged PFRC, corresponding to a reduction of approximately 21.98%. A two-tailed independent-samples Welch’s t-test confirmed that this reduction was statistically significant, with a mean difference of 15.55 MPa (95% confidence interval: 13.01–18.08 MPa, p < 0.001). The reduction remained statistically significant after Holm correction across the four prespecified mechanical-property comparisons (adjusted p < 0.001). The similar standard deviations of the two groups indicate that the apparent short-beam shear strength retained a relatively narrow distribution after field service, despite the pronounced reduction in its mean value.

**Table 8:** Apparent short-beam shear strength results and statistical comparison of pristine and service-aged PFRC.

Specimen state	Mean ± SD (MPa)	Mean difference (MPa)	95% CI of mean difference (MPa)	t	df	Unadjusted p-value	Holm-adjusted p-value
pristine	70.76 ± 1.77	15.548	13.01–18.08	14.143	7.992	<0.001	<0.001
service-aged	55.21 ± 1.71						

**Fig 14:**
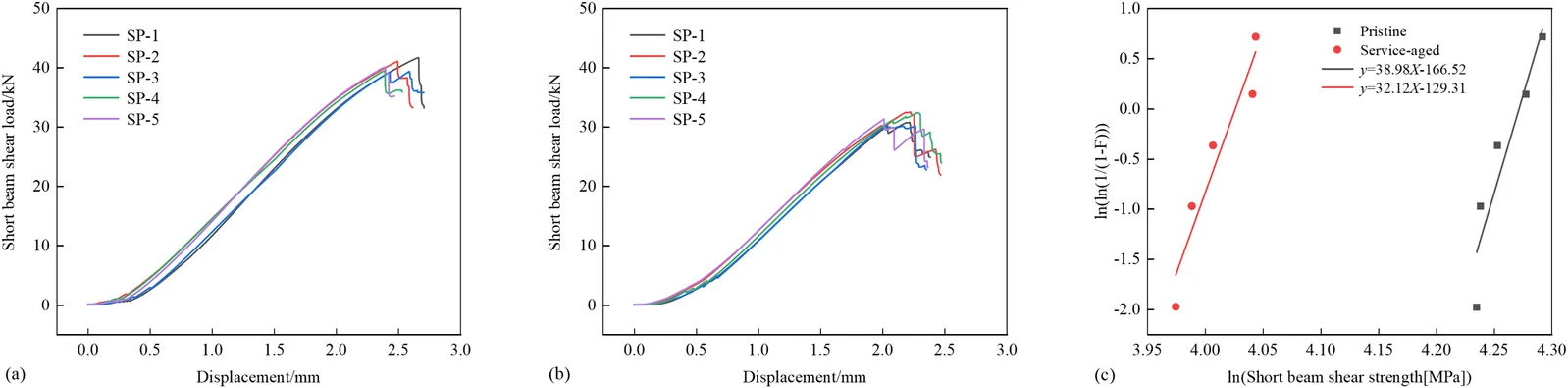
Apparent horizontal shear response and Weibull analysis of pristine and service-aged PFRC: (a) load–displacement curves of pristine specimens; (b) load–displacement curves of service-aged specimens; (c) Weibull regression plots.

The Weibull results are presented in Fig 14(c) and Table 9. The LS estimates of *m* were 39.00 for the pristine PFRC and 32.10 for the service-aged PFRC, with broad 95% bootstrap confidence intervals of 15.47–99.53 and 13.11–83.60, respectively. The corresponding *σ*_0_ decreased from 71.64 to 56.04 MPa. MLE gave somewhat higher *m* values of 47.67 and 41.49, while the scale parameters were 71.56 and 55.96 MPa, closely matching the LS estimates. Compared with the other loading modes, the change in the shape parameter was relatively modest, whereas the reduction in *σ*_0_ was pronounced and nearly independent of the fitting method. Together with the similar experimental standard deviations, this indicates that field service mainly reduced the apparent short-beam shear-strength level without a marked increase in strength dispersion.

**Table 9:** Weibull parameters obtained using median-rank least-squares fitting and maximum likelihood estimation for apparent short-beam shear strength.

Specimen state	Experimental mean (MPa)	LS *m*	95% bootstrap CI of LS *m*	MLE *m*	LS *σ*_0_ (MPa)	95% bootstrap CI of LS *σ*_0_ (MPa)	MLE *σ*_0_ (MPa)	LS *R*^2^
Pristine	70.76	39	15.47–99.53	47.67	71.64	69.74–73.23	71.56	0.874
Service-aged	55.21	32.1	13.11–83.6	41.49	56.04	54.22–57.54	55.96	0.918

The failure morphology under apparent horizontal shear loading also changed after field service. The pristine PFRC exhibited localized interfacial delamination and an isolated interfacial crack, while the surrounding material retained a relatively compact structure (Fig 15(a, b)). In contrast, the service-aged specimens exhibited more extensive matrix damage, fiber–matrix debonding, and multiple interlaminar cracks within the loading region (Fig 15(c, d)). The wider distribution of matrix cracking, interfacial debonding, and delamination after service further indicates a transition toward more spatially distributed and interface-controlled shear damage within the affected region. These changes are consistent with weakened interlaminar cohesion and reduced shear-transfer capability after field service. Importantly, the similar standard deviations of the two groups indicate that the dominant effect was a reduction in shear-strength level rather than a substantial increase in strength dispersion.

**Fig 15:**
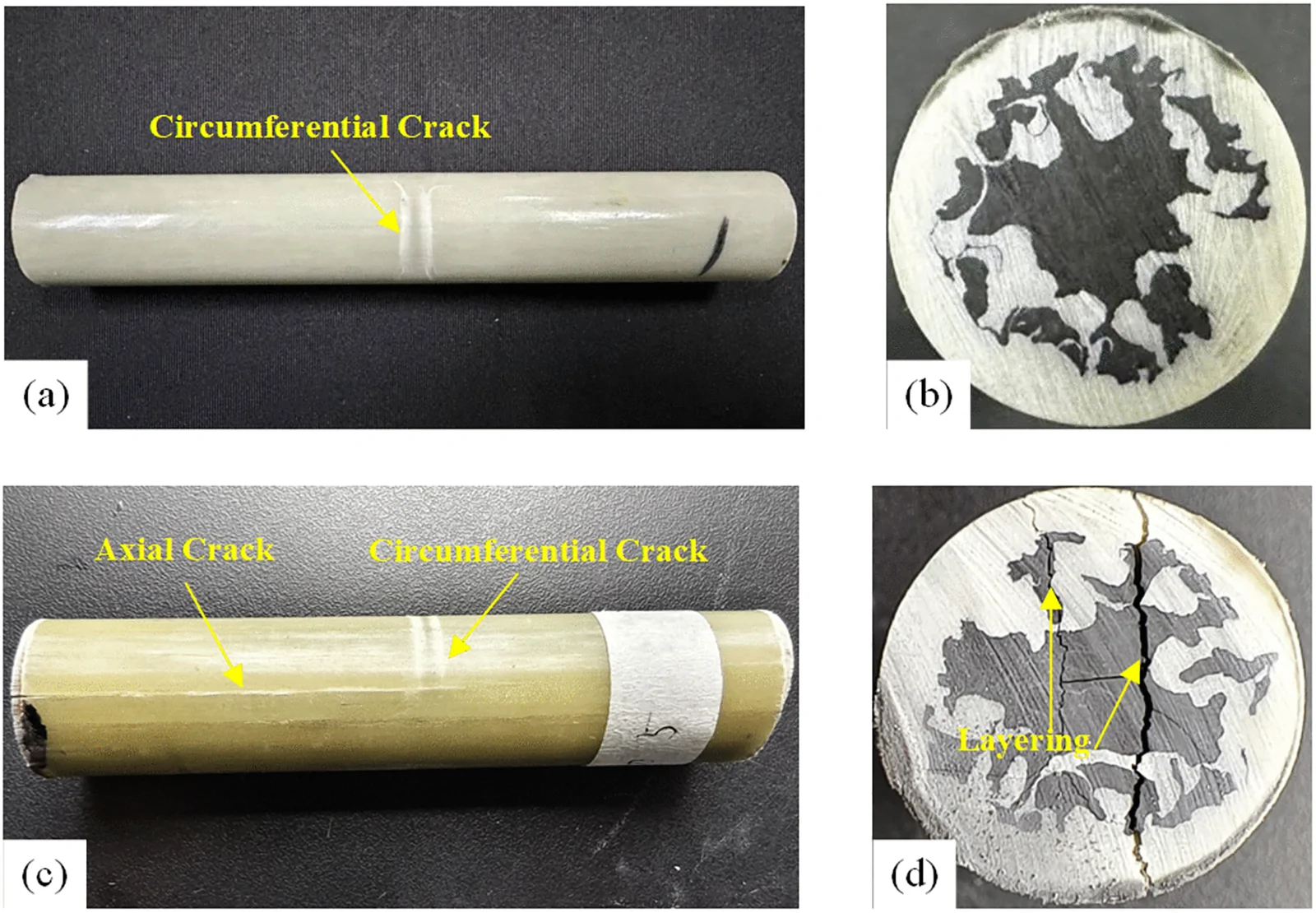
Macroscopic failure morphologies of pristine and service-aged PFRC: (a) pristine PFRC; (b) cross-section of pristine PFRC; (c) service-aged PFRC; (d) cross-section of service-aged PFRC.

#### 3.1.5. Comparison of mechanical degradation with previous field studies.

The mechanical degradation of the field-serviced PFRC exhibited a clear dependence on loading mode. After 435 days of service, the tensile strength decreased by 10.02%, whereas the flexural and apparent short-beam shear strengths decreased by 19.92% and 21.98%, respectively. The smaller reduction in tensile strength reflects the continued load-carrying contribution of the longitudinal fibers, while the larger reductions in flexural and apparent short-beam shear strengths indicate greater deterioration of the resin matrix, fiber–matrix interfaces, and internal hybrid transition region. Therefore, the field exposure did not produce a uniform reduction in all mechanical properties, but preferentially affected properties that depend strongly on transverse constraint and interfacial stress transfer.

Related field investigations on pultruded carbon/glass hybrid rods have also reported pronounced degradation in interface-sensitive properties. A maximum short-beam shear strength reduction of 18.92% was observed in a 22 mm hybrid rod exposed in an actual oil well for up to 553 days, together with maximum interface shear strength reductions of 28.24%, 16.88%, and 36.07% for the carbon-fiber/resin, glass-fiber/resin, and carbon-core/glass-shell interfaces, respectively [14]. In a related study on a 19 mm rod, the final short-beam shear strength reductions after 606 days were 2.9% and 10.1% under two different field conditions [13], although larger reductions occurred during the earlier exposure stage before partial recovery associated with resin post-curing. Collectively, these studies indicate that field degradation can be non-monotonic because the measured response may reflect the competition between moisture-induced deterioration and continued resin curing [15].

Direct quantitative equivalence between the present results and previous field studies should be avoided because of differences in composite architecture and service conditions. The present PFRC comprises an outer glass-fiber layer, an intermediate carbon/glass hybrid transition layer, and an inner carbon-fiber core, whereas the previously reported rods mainly adopted a two-layer carbon-core/glass-shell configuration. This architectural difference, together with variations in resin formulation, curing state, layer dimensions, fluid chemistry, temperature, pressure, cyclic loading, and sampling time, may alter moisture-transport pathways, interfacial stress transfer, and crack-propagation behavior, thereby contributing to the different degradation magnitudes. Nevertheless, both the present and previous field results indicate that interface-rich transition regions are critical to long-term shear and flexural performance.

### 3.2. Microscopic morphology analysis

#### 3.2.1 Field failure splitting fracture SEM.

To characterize the microscopic damage features associated with the splitting failure, SEM specimens were prepared from the PFRC that fractured after 435 days of service. Several cylindrical specimens with a height of 10 mm were sectioned from the failure region of the split PFRC, as shown in Fig 16. These specimens were sequentially subjected to ultrasonic cleaning for 15 minutes, immersion in acetone for 1 minute, and rinsing with absolute ethanol, followed by blow-drying. SEM observations focused on the split cross-section, with a primary emphasis on analyzing the longitudinal fiber distribution and defect morphology.

**Fig 16:**
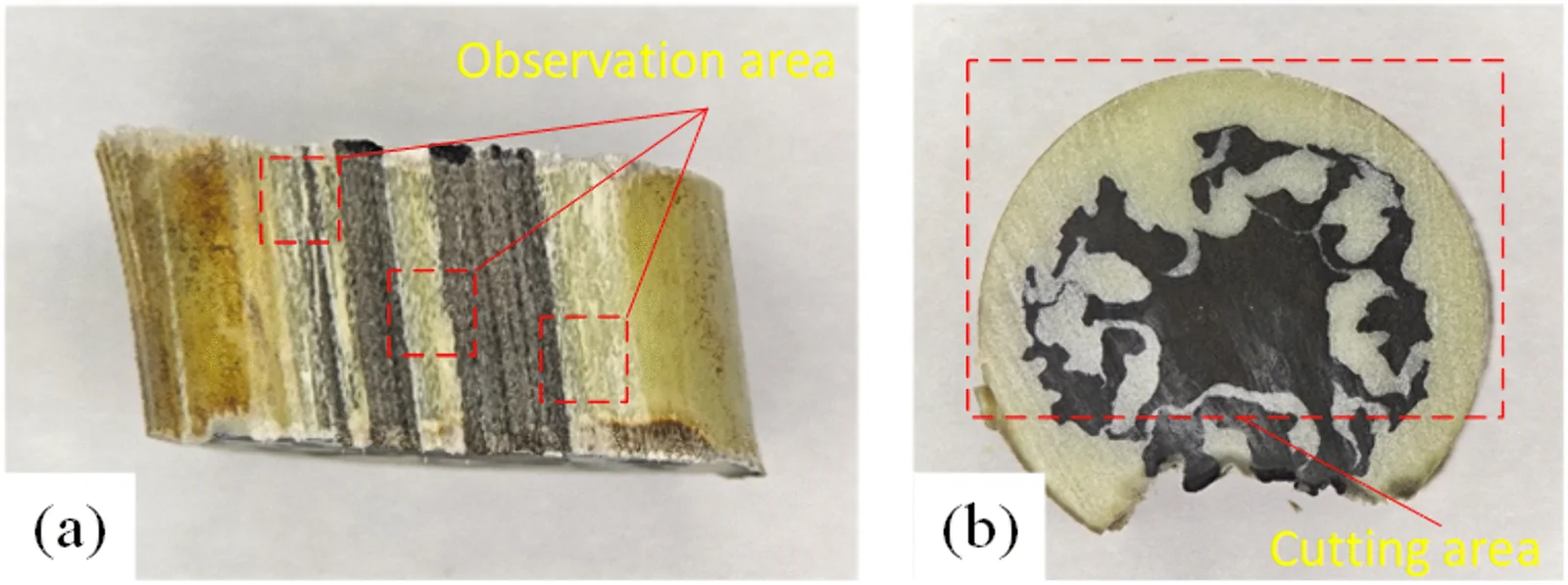
Specimen of split part of service rod. (a) Observation area, (b) Cutting area.

As illustrated in Fig 17, the split cross-section of the service-aged PFRC exhibited multiscale damage involving the carbon-fiber region and the carbon/glass hybrid transition region. In the carbon-fiber region shown in Fig 17(a), fiber breakage, matrix fragmentation, and fiber–matrix interfacial debonding were observed. Localized crater-like features and fiber pull-out grooves were also present, while the exposed carbon-fiber surfaces showed little residual resin adhesion. These features are consistent with deterioration of the fiber–matrix interfacial bonding after long-term field service.

**Fig 17:**
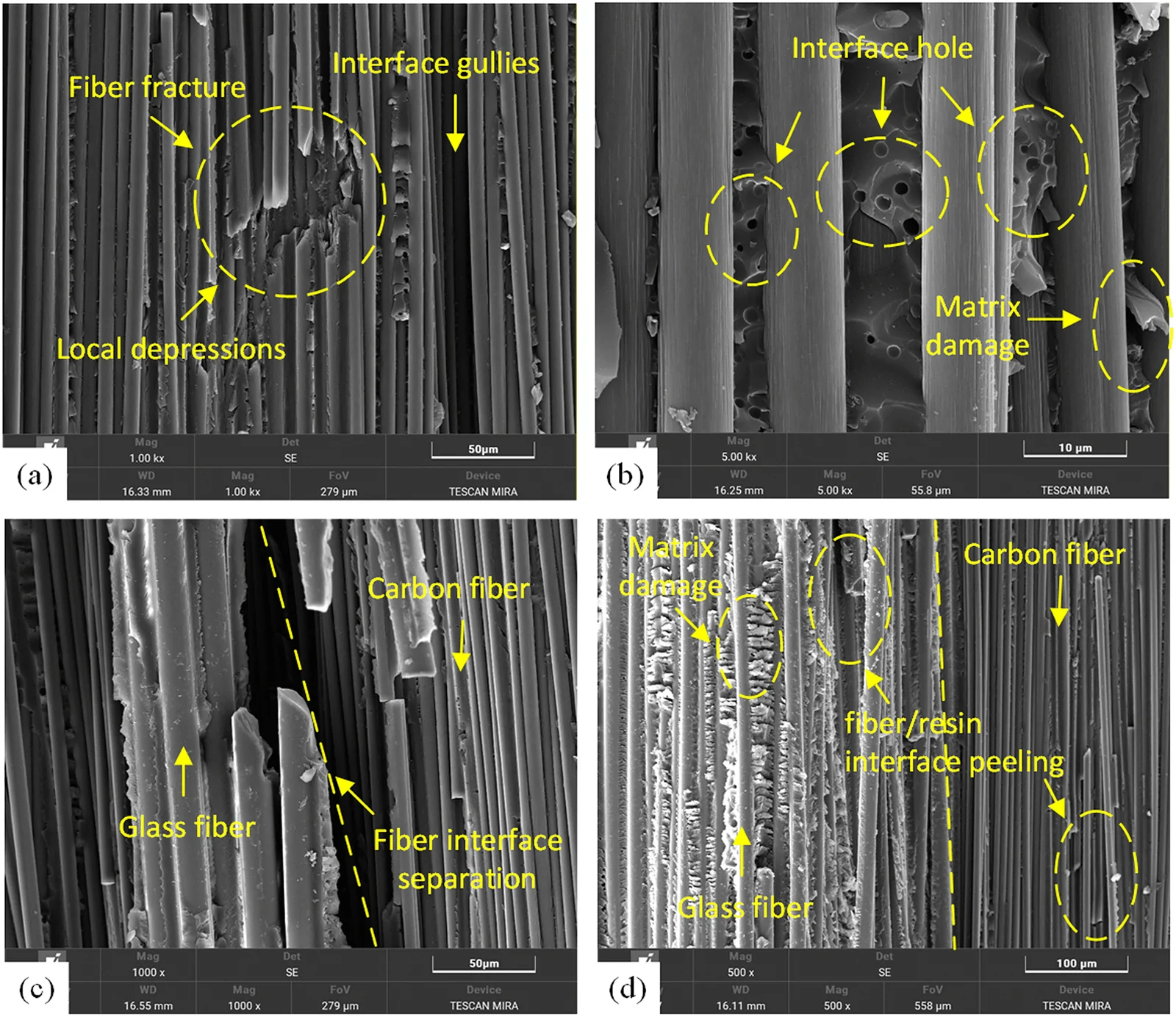
Microscopic morphology of the service-aged PFRC after longitudinal splitting. (a) Carbon fiber region, (b) Carbon/glass fiber boundary, (c) Separation of the hybrid interface, (d) Complex damage at the hybrid interface.

At the carbon/glass hybrid transition region, more pronounced interfacial damage was observed. Fig 17(b) shows matrix fragmentation and interfacial voids near the boundary between the carbon-fiber-rich and glass-fiber-rich regions. Fig 17(c) further shows local separation of the carbon/glass hybrid interface, indicating substantial loss of interfacial continuity. In Fig 17(d), more complex damage is evident, including resin-matrix crushing on the glass-fiber side together with interfacial delamination and separation across the hybrid boundary. Taken together, the damage features in Fig 17(b–d) indicate that the carbon/glass hybrid transition region was particularly susceptible to accumulated matrix and interfacial degradation during field service.

Because the macroscopic longitudinal splitting was observed during retrieval and drum winding rather than during downhole operation, the SEM observations alone cannot directly identify the actual field stress state responsible for failure. Nevertheless, the pronounced interfacial separation and delamination observed in Fig 17(b–d) are consistent with a weakened hybrid interface that could be more susceptible to crack propagation under additional bending deformation. The stiffness mismatch between the carbon- and glass-fiber-rich regions may contribute to deformation incompatibility and elevated local interfacial stresses during bending. Therefore, the observed morphology is interpreted as supporting evidence that pre-existing matrix and interfacial damage accumulated during long-term service may have propagated and coalesced during retrieval-induced bending, thereby contributing to the observed longitudinal splitting.

#### 3.2.2 Micromorphology of tensile fracture.

In accordance with the GB/T 13096−2008 standard, tensile tests were conducted on the PFRC before and after service. To observe the internal failure morphology, 20 mm-long SEM specimens were prepared, as shown in Fig 18. The specimen shown in Fig 18(a), derived from the pristine PFRC, was sectioned from the central crack propagation region, specifically targeting the site that exhibited the most representative failure characteristics. The specimen shown in Fig 18(b), obtained from the service-aged PFRC, was extracted from the terminal end of the burst region.

**Fig 18:**
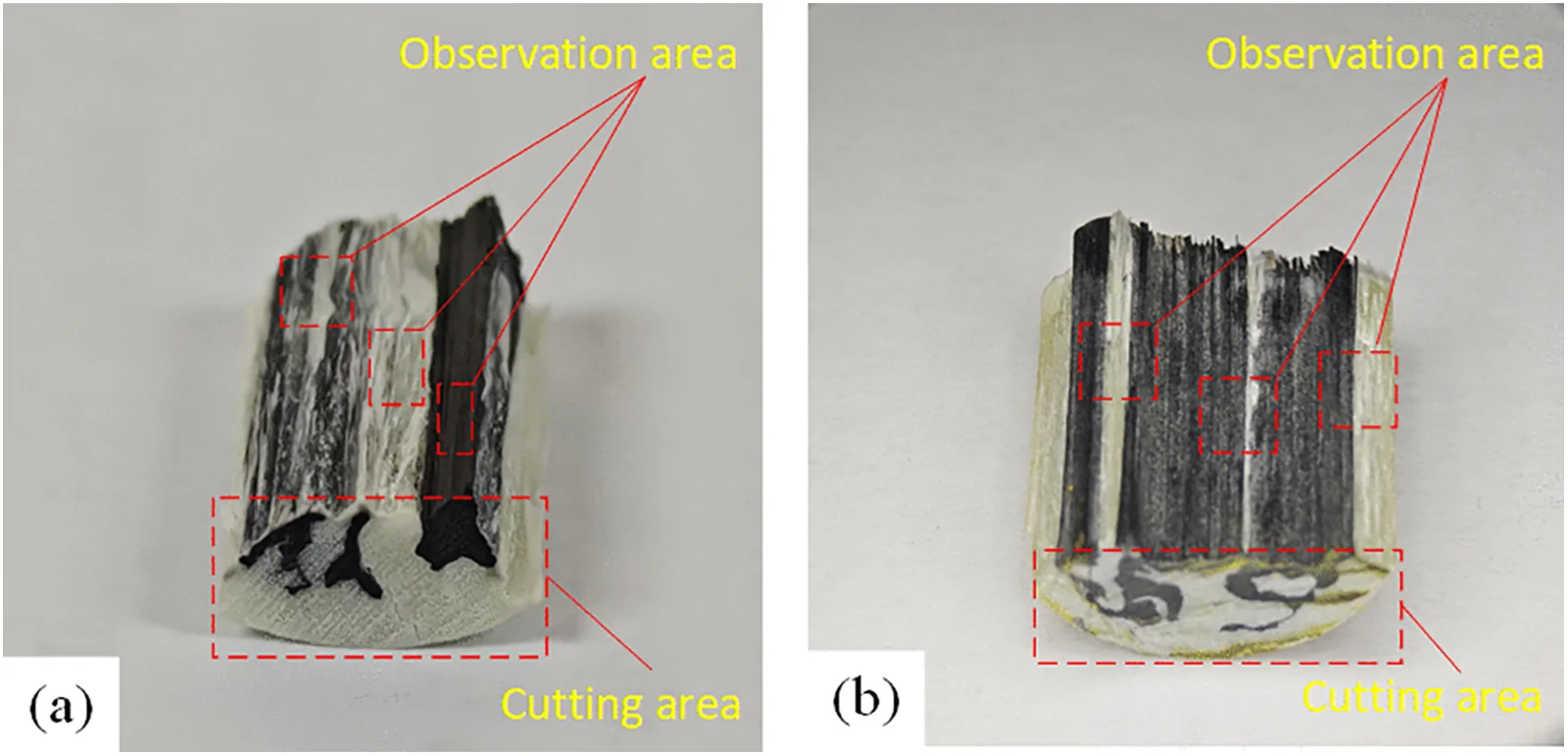
SEM specimens of PFRC before and after service. (a) Pristine PFRC, (b) Service-aged PFRC.

Fig 19 illustrates the microscopic tensile damage mechanisms of the pristine PFRC and the 435-day service-aged PFRC. Both specimens exhibited fiber fracture, matrix cracking, and fiber–matrix interfacial damage, but the extent and distribution of these features differed markedly after field service. In the pristine PFRC, fractured fibers remained surrounded by relatively continuous resin, indicating effective interfacial constraint during tensile loading. In contrast, the service-aged PFRC exhibited smoother exposed carbon-fiber surfaces, reduced resin adhesion, extensive matrix fragmentation, and more pronounced interfacial separation, as shown in Fig 19(c, d). These observations are consistent with weakened fiber–matrix adhesion and reduced efficiency of axial load transfer after long-term service.

**Fig 19:**
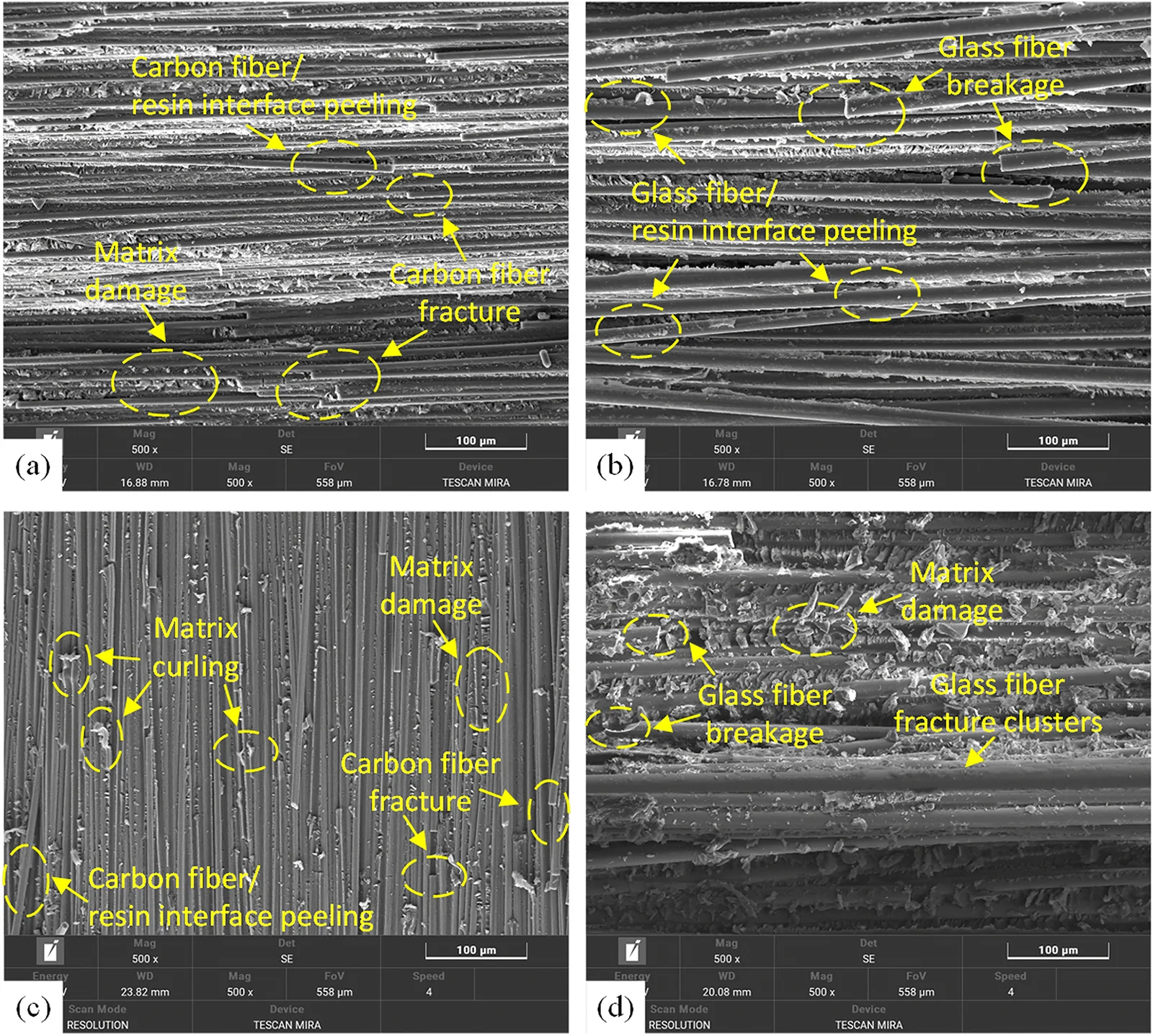
Microscopic tensile damage morphology of pristine and service-aged PFRC: (a) carbon-fiber region; (b) glass-fiber region; (c) carbon-fiber/matrix interfacial region; (d) outer glass-fiber region.

Quantitative ImageJ analysis further supported these morphological observations. As summarized in Table 10, for resin coverage analysis, one whole-field ROI was defined for each SEM field, and the exposed-fiber areas were measured within the corresponding ROI. A total of 50, 42, and 21 exposed-fiber area measurements were obtained from the three pristine SEM fields, respectively, whereas 103, 25, and 19 measurements were obtained from the three service-aged fields. The average resin coverage ratio on the carbon-fiber surfaces decreased from 67.89% ± 4.49% in the pristine PFRC to 60.65% ± 5.48% after field service. For carbon-fiber pull-out length, nine ROIs were analyzed for each condition, yielding 18, 19, and 16 individual measurements in the pristine fields and 16, 15, and 15 measurements in the service-aged fields, respectively. The mean carbon-fiber pull-out length increased from 93.80 ± 44.96μm to 133.50 ± 74.55μm. The reduction in resin coverage provides a quantitative indication of reduced resin adhesion after service. Concurrently, the pronounced increase in both the mean pull-out length and its standard deviation reflects greater interfacial slip and increased morphological variability during failure. Together with the observed matrix fragmentation and interfacial debonding, these results are consistent with a transition from predominantly cohesive matrix fracture toward more interface-controlled tensile damage in the service-aged PFRC.

**Table 10:** Quantitative characterization of tensile fracture morphology and interfacial bonding state.

Sucker rod structure type	Average resin coverage ratio on fiber surface (%)	Average carbon fiber pull-out (exposed) length (μm)	Tensile strength (MPa)
Pristine PFRC	67.89% ± 4.49%	93.80 ± 44.96	1283.27
Service-aged PFRC	60.65% ± 5.48%	133.50 ± 74.55	1154.68

#### 3.2.3 Micromorphology of compression failure.

In accordance with the GB/T 1448–2005 standard, compression tests were conducted on the PFRC before and after service. To observe the internal failure morphology, 20 mm-long SEM specimens were prepared, as shown in Fig 20. The macroscopic compressive failure modes of both specimens appeared similar, characterized by longitudinal cracks propagating downwards along the axial direction of the PFRC, accompanied by matrix crushing. To further examine the internal failure characteristics, the specimens were sectioned along these longitudinal cracks. Intact structures from the central region of the crack propagation path were then extracted for SEM inspection, as depicted in Fig 20(a, b).

**Fig 20:**
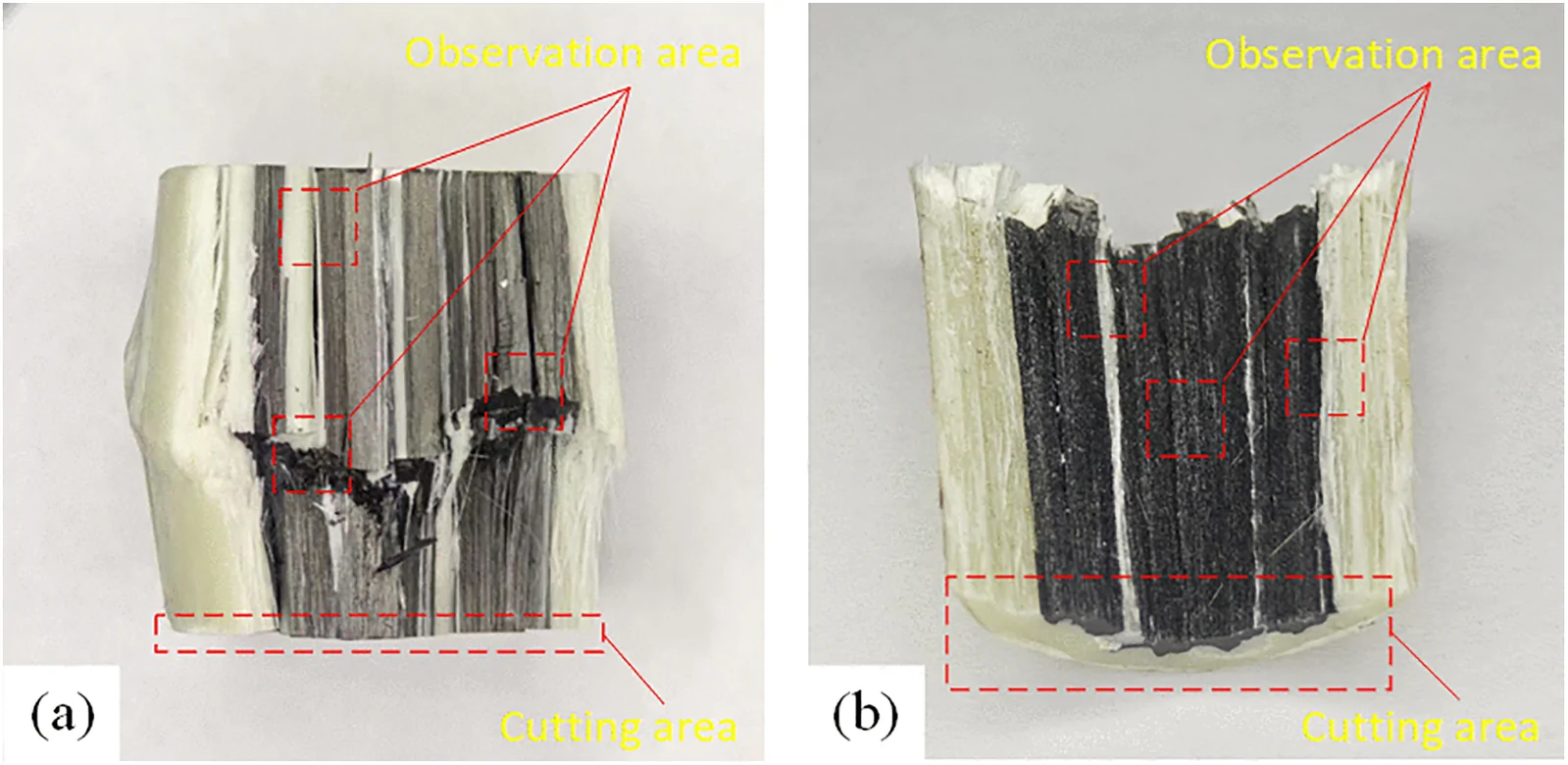
SEM specimens of PFRC before and after service. (a) Pristine PFRC, (b) Service-aged PFRC.

Fig 21 illustrates the compressive damage micromechanisms of the pristine PFRC and the 435-day service-aged PFRC. The pristine PFRC exhibited relatively localized damage characterized by matrix crushing and fiber instability, indicating that the surrounding matrix and interfaces still provided effective lateral constraint to the fiber bundles. In contrast, the service-aged PFRC showed more dispersed longitudinal splitting along weakened regions, accompanied by matrix fragmentation and interfacial cracking. These observations suggest that long-term service reduced the lateral constraint on the reinforcing fibers and increased the sensitivity of compressive failure to pre-existing defects.

**Fig 21:**
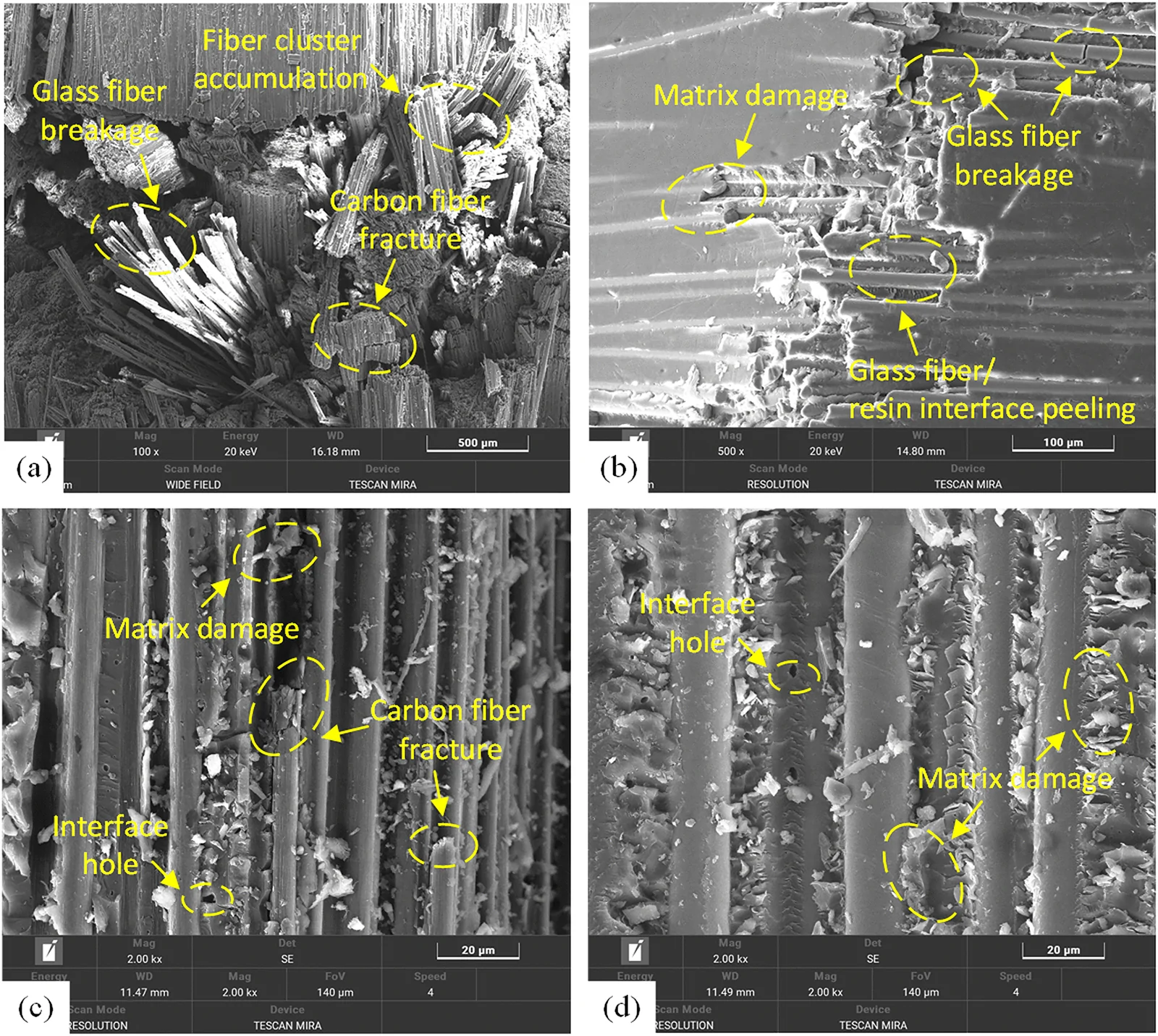
Microscopic compressive damage morphology of pristine and service-aged PFRC. (a) Localized concentrated fracture region, (b) Surface glass fiber-resin interfacial region, (c) Longitudinal split cross-section, (d) Minute void defects in resin matrix.

Quantitative image analysis further confirmed the change in compressive damage morphology. As summarized in Table 11, for the average opening width of the longitudinal main crack, nine ROIs were selected across the three SEM fields for the pristine PFRC, yielding 50, 60, and 39 individual crack-width measurements in the three respective fields. For the service-aged PFRC, six ROIs were selected across the three SEM fields, yielding 53, 66, and 24 individual measurements, respectively. The average opening width of the principal longitudinal crack increased from 1.37 ± 0.49 mm in the pristine PFRC to 3.51 ± 2.35 mm after field service, indicating a pronounced widening of the main longitudinal crack accompanied by increased morphological variability. For the average total length of cross-sectional radial cracks, one ROI was assigned to each SEM field, and the total crack length was obtained by summing the lengths of all clearly identifiable radial-crack segments within each field. In the pristine PFRC, 24, 35, and 69 radial-crack segments were identified and measured in the three respective SEM fields. In the service-aged PFRC, 31, 39, and 64 radial-crack segments were measured in the corresponding fields. The resulting average total length of cross-sectional radial cracks increased from 59.61 ± 5.79 mm in the pristine PFRC to 76.08 ± 18.46 mm after field service. The substantially larger standard deviation of the longitudinal crack width and radial-crack length in the service-aged PFRC further indicates increased irregularity and spatial heterogeneity of the fracture morphology.

**Table 11:** Quantitative characterization of compressive crack evolution and structural integrity.

Sucker rod structure type	Average opening width of the longitudinal main crack (mm)	Average total length of cross-sectional radial cracks (mm)	Compressive strength (MPa)
Pristine PFRC	1.37 ± 0.49	59.61 ± 5.79	554.70
Service-aged PFRC	3.51 ± 2.35	76.08 ± 18.46	542.21

These changes indicate more extensive and highly variable longitudinal and radial crack development in the service-aged PFRC. Although the mean compressive strength decreased by only 2.25%, its coefficient of variation increased markedly from 2.42% to 18.88%, indicating that the more notable effect of field service was an increase in the heterogeneity and variability of compressive failure rather than a substantial reduction in mean compressive strength.

#### 3.2.4 Micromorphology of bending failure.

In accordance with the GB/T 13096−2008 standard, bending tests were performed on the PFRC before and after service. To observe the internal failure morphology, 20 mm-long SEM specimens were prepared, as shown in Fig 22. The tested pristine PFRC exhibited a localized indentation region in the vicinity of the loading point, while its overall structure remained intact without evident cracking. Given the absence of overt splitting failure, the pristine PFRC was sectioned at both ends of the indentation region. During this preparation process, latent internal splitting cracks caused the sample to separate into two halves. One of these halves was subsequently utilized for the SEM inspection shown in Fig 22(a). Conversely, the service-aged PFRC shown in Fig 22(b) was extracted directly from the central portion of the split region.

**Fig 22:**
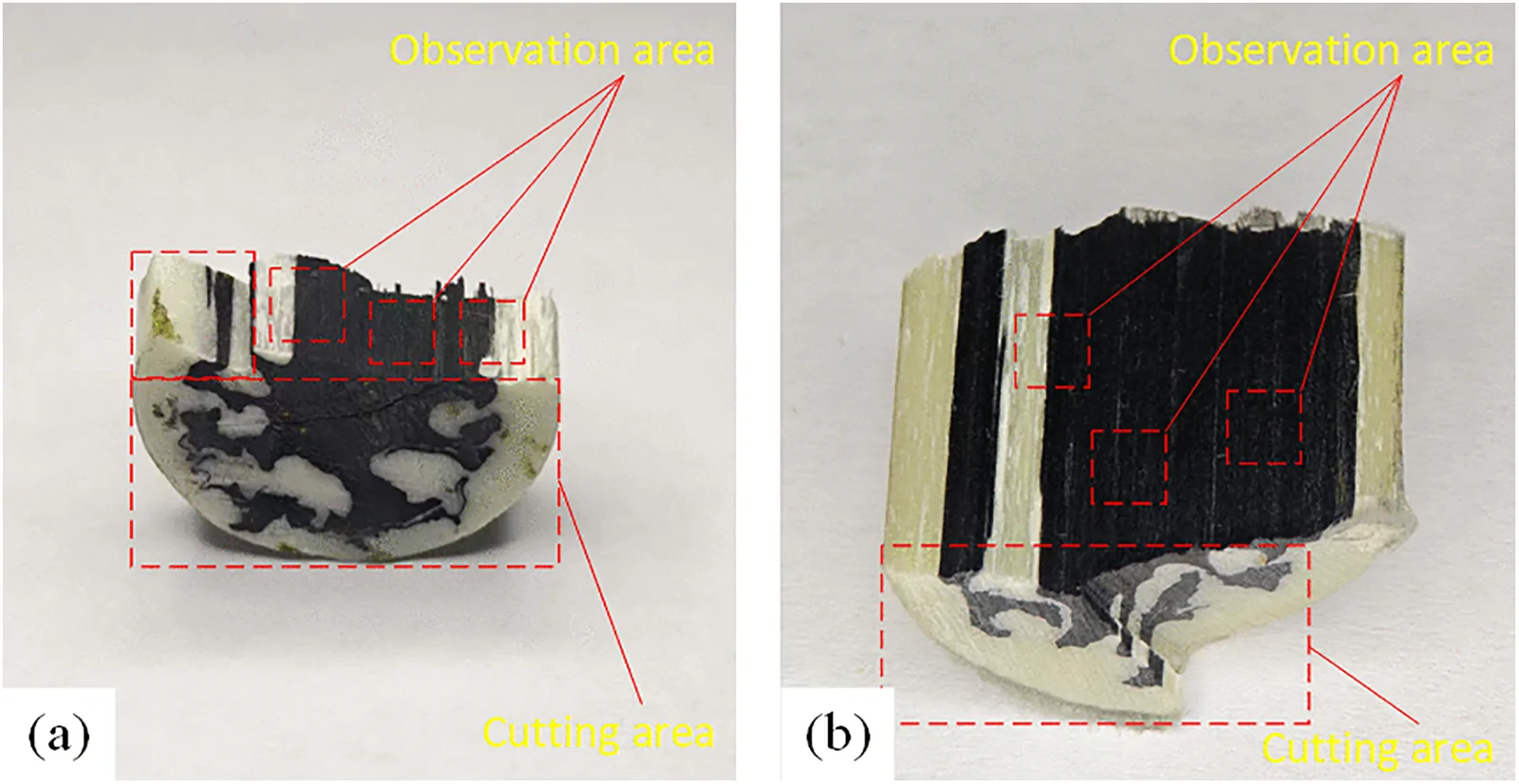
SEM specimens of PFRC before and after service. (a) Pristine PFRC, (b) Service-aged PFRC.

Fig 23 illustrates the microscopic flexural damage characteristics of the pristine and 435-day service-aged PFRC. In the pristine PFRC, fractured carbon fibers remained relatively well embedded in the surrounding resin, whereas the glass-fiber fracture surfaces exhibited comparatively less resin adhesion. After field service, both regions showed reduced resin attachment, more pronounced fiber–matrix debonding, and interfacial crack propagation along the carbon/glass transition region. These observations indicate that long-term service weakened the interfacial integrity required for coordinated deformation between the carbon- and glass-fiber regions during bending.

**Fig 23:**
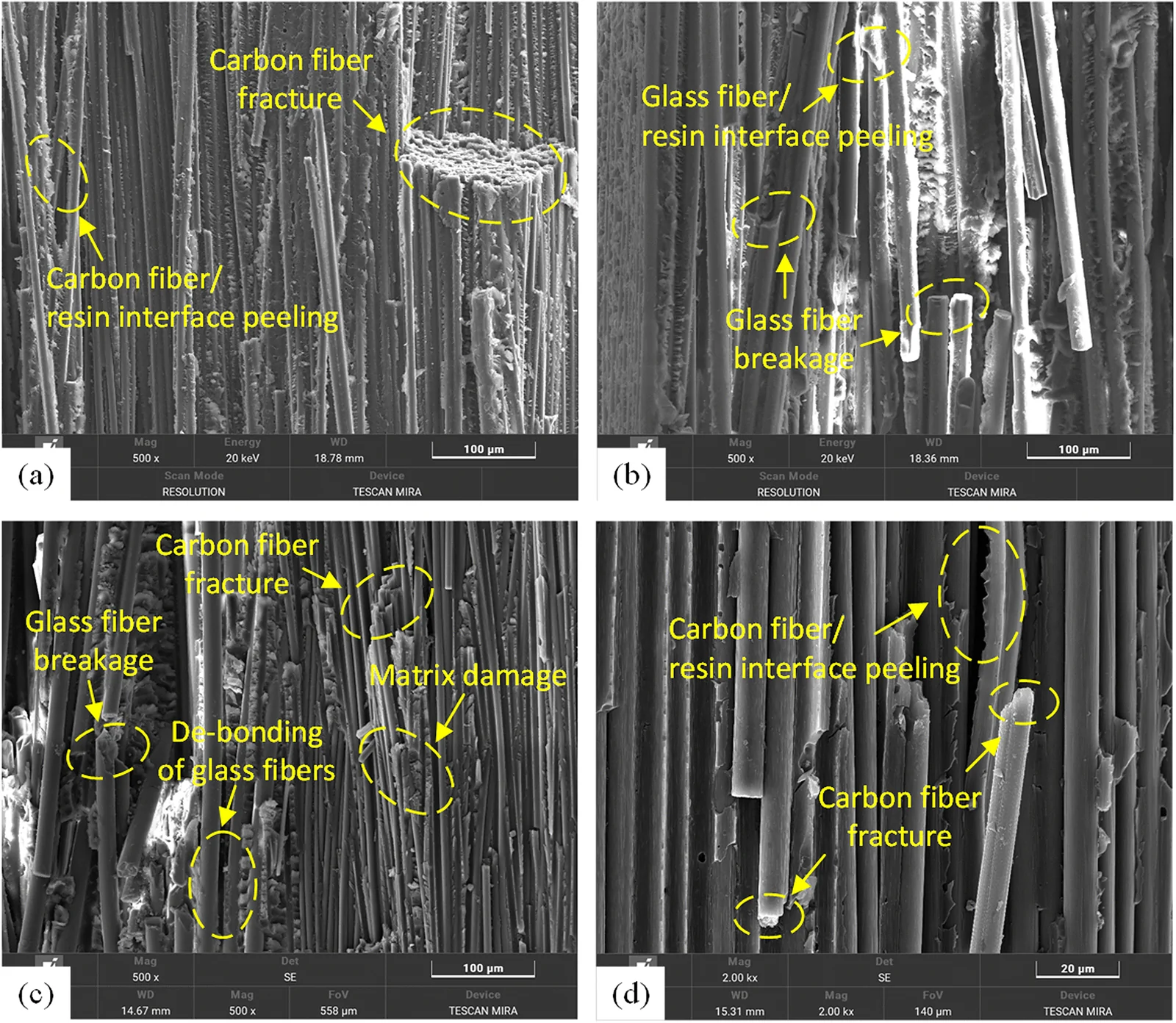
Microscopic flexural damage morphology of pristine and service-aged PFRC. (a) Fractured surfaces of the carbon fibers, (b) Fractured surfaces of the glass fibers, (c) Carbon fiber interface, (d) Glass fiber interface.

Quantitative ImageJ analysis further supported this change in flexural damage morphology. As summarized in Table 12, for carbon-fiber pull-out length, eight ROIs were analyzed for the pristine PFRC and nine ROIs for the service-aged PFRC, yielding 6, 8, and 31 individual measurements in the three pristine SEM fields and 7, 14, and 17 measurements in the three service-aged fields, respectively. The mean carbon-fiber pull-out length exhibited a moderate increase from 116.05 ± 44.20 μm to 127.50 ± 59.06 μm after field service. For interfacial debonding-gap width, ten ROIs were analyzed for the pristine PFRC and eight for the service-aged PFRC, with 50, 45, and 99 individual gap-width measurements obtained from the three pristine fields and 44, 80, and 57 measurements from the three service-aged fields, respectively. The mean interfacial debonding-gap width remained nearly unchanged from 10.76 ± 3.84 μm to 10.79 ± 4.12 μm. This observable increase in pull-out length and its broader variability, together with the reduced resin adhesion and interfacial cracking observed by SEM, is consistent with weakened interfacial bonding and less effective coordinated deformation between the carbon- and glass-fiber regions. The relatively stable mean debonding-gap width indicates that the dominant morphological change was associated with enhanced fiber extraction rather than a uniform widening of the interfacial gap. These morphological changes are also consistent with the 19.92% reduction in flexural strength after field service.

**Table 12:** Quantitative characterization of interlaminar damage under flexural loading.

Sucker rod structure type	Average interfacial debonding gap width (μm)	Average carbon fiber pull-out (exposed) length (μm)	Flexural strength (MPa)
Pristine PFRC	10.76 ± 3.84	116.05 ± 44.20	1298.18
Service-aged PFRC	10.79 ± 4.12	127.50 ± 59.06	1039.56

#### 3.2.5 Micromorphology of shear failure.

In accordance with the GB/T 13096−2008 standard, shear tests were conducted on the PFRC before and after service. To observe the internal failure morphology, SEM specimens with a length of 20 mm were prepared, as shown in Fig 24. During testing, surface cracks emerged on both the pristine and service-aged PFRC. Consequently, these specimens were sectioned at locations remote from both ends of the indentation. During the preparation process, propagating internal cracks caused the samples to separate into two halves; one half of each was subsequently selected for the SEM inspection shown in Fig 24(a, b).

**Fig 24:**
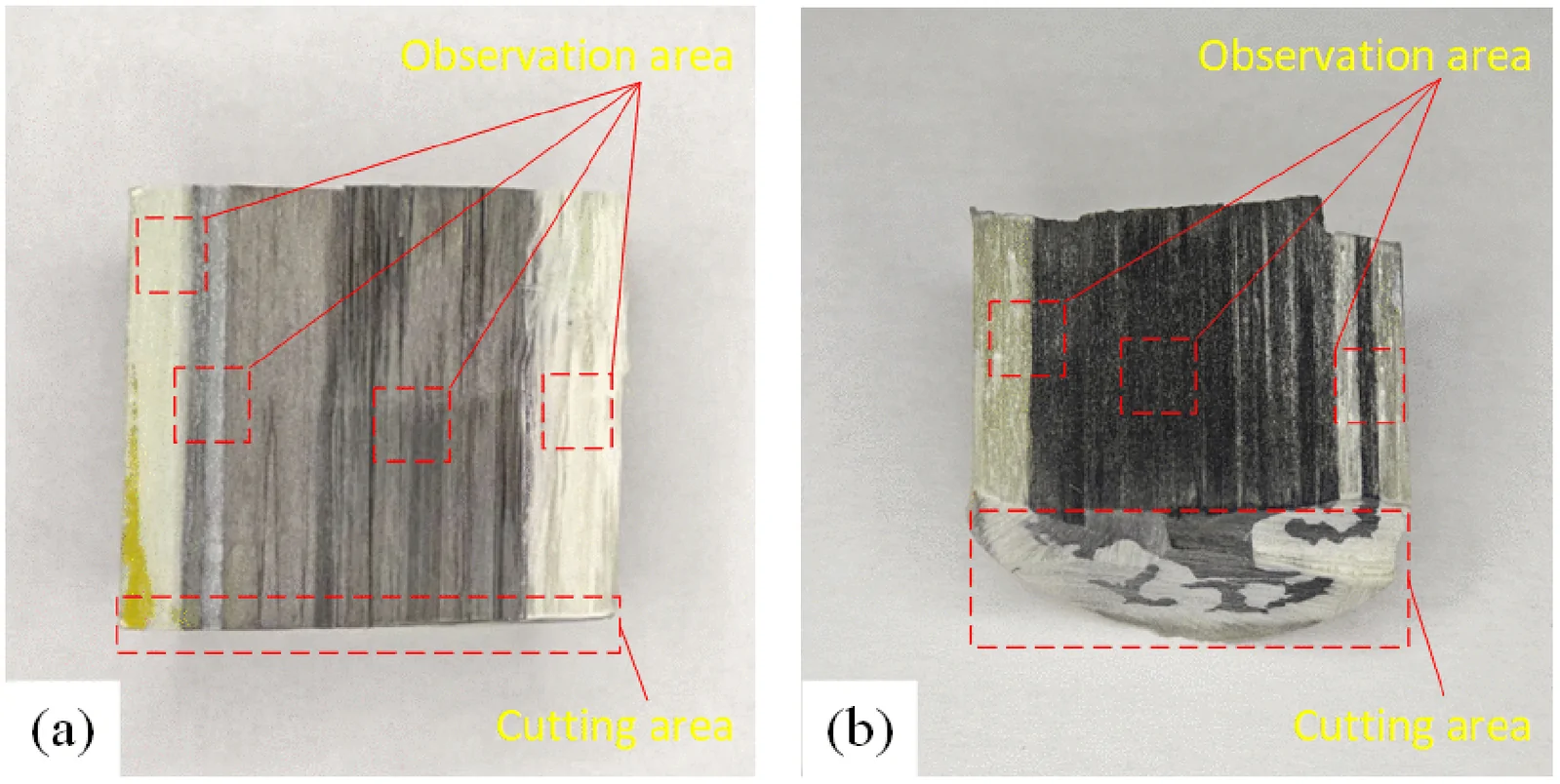
SEM specimens of PFRC before and after service. (a) pristine rod, (b) service-aged rod.

Fig 25 illustrates the microscopic shear damage characteristics of the pristine and 435-day service-aged PFRC. In the pristine PFRC, fiber fracture and matrix cracking remained relatively localized, and substantial resin adhesion was retained on the fiber surfaces, indicating comparatively intact fiber–matrix interfaces. In contrast, the service-aged PFRC exhibited more extensive fiber–matrix debonding, fragmented resin debris, smoother exposed fibers, and longitudinal interfacial cracking. These observations indicate that long-term service weakened the integrity of the resin-rich interfacial regions and reduced their ability to sustain shear transfer during short-beam loading.

**Fig 25:**
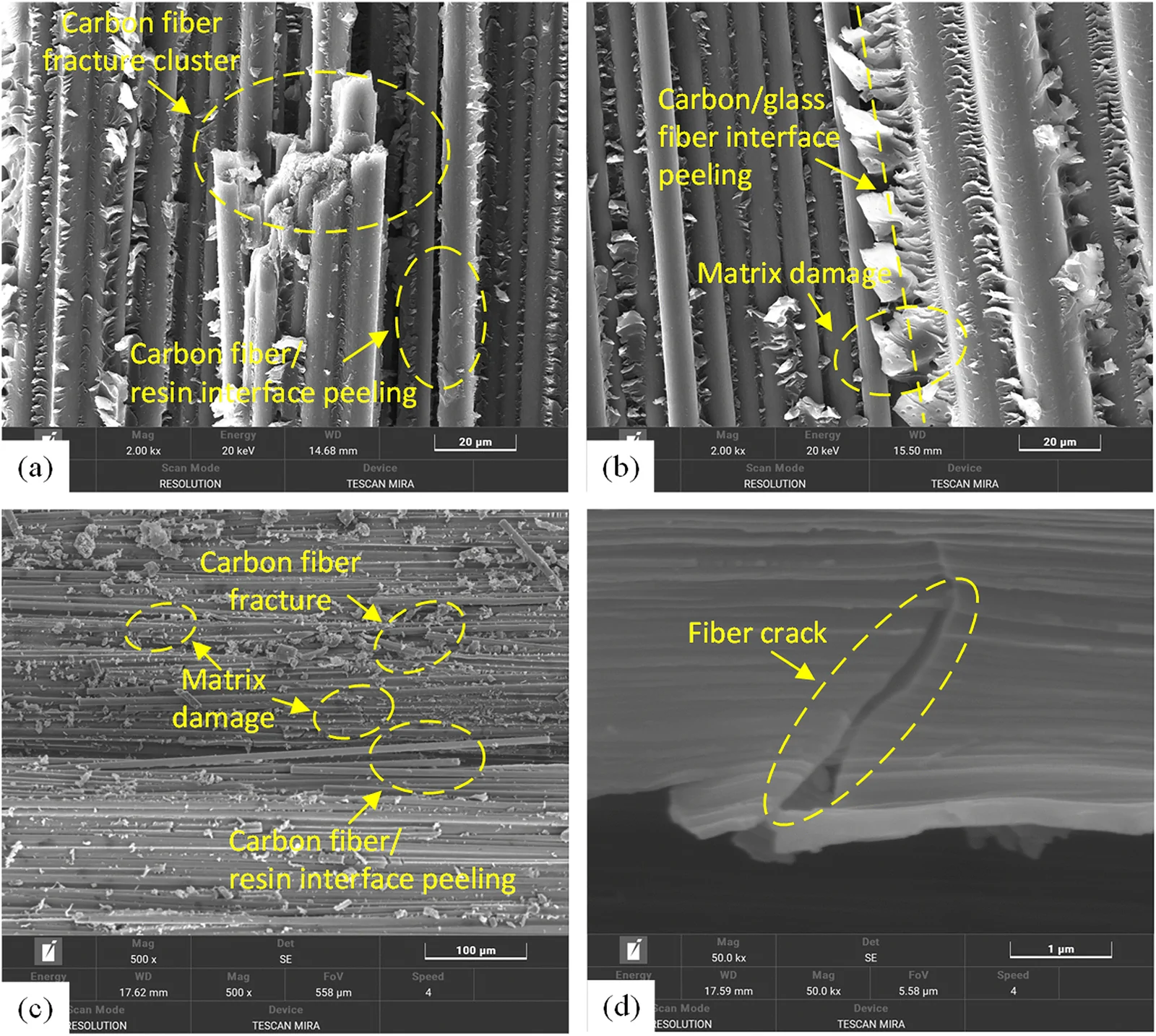
Microscopic shear damage morphology of pristine and service-aged PFRC. (a) Carbon fiber fracture zone, (b) Carbon/glass fiber transition region, (c) Carbon fiber/matrix debonding zone, (d) Longitudinal crack defects within fibers.

Quantitative ImageJ analysis further supported this change in shear damage morphology. As summarized in Table 13, for carbon-fiber pull-out length, seven ROIs were analyzed for both the pristine and service-aged PFRC, yielding 8, 9, and 26 individual measurements in the pristine fields and 7, 20, and 21 measurements in the service-aged fields, respectively. The mean carbon-fiber pull-out length increased from 100.58 ± 73.99 μm in the pristine PFRC to 206.57 ± 120.58 μm after field service. For interfacial debonding-gap width, eleven ROIs were analyzed for the pristine PFRC and six for the service-aged PFRC, yielding 10, 95, and 145 individual gap-width measurements in the pristine fields and 12, 63, and 39 measurements in the service-aged fields, respectively. The mean interfacial debonding-gap width increased from 2.92 ± 1.36 μm to 9.79 ± 6.85 μm. These correspond to approximately 2.1-fold and 3.4-fold increases in the mean values, respectively, accompanied by a substantial broadening of their standard deviations. Together with the reduced resin adhesion observed by SEM, these changes are consistent with substantial weakening of the fiber–matrix interface and a transition toward more interface-controlled shear damage. The quantitative morphological changes are also consistent with the 21.98% reduction in apparent short-beam shear strength after field service.

**Table 13:** Quantitative characterization of shear-induced interfacial degradation.

Sucker rod structure type	Average interfacial debonding gap width (μm)	Average carbon fiber pull-out (exposed) length (μm)	Apparent Short-beam shear strength (MPa)
Pristine PFRC	2.92 ± 1.36	100.58 ± 73.99	70.76
Service-aged PFRC	9.79 ± 6.85	206.57 ± 120.58	55.21

### 3.3. DMA results

DMA was performed to compare the thermomechanical response of the carbon-fiber core before and after long-term field service. As shown in Fig 26(a, c), *T*_*g*_ increased slightly from 159.5 °C for the pristine PFRC to 164.76 °C for the service-aged PFRC, whereas the peak tan *δ* value decreased from 0.23 to 0.14. Concurrently, as shown in Fig 26 (b), the storage modulus decreased markedly from 78.4GPa to 51.5GPa, corresponding to a reduction of 34.3%. This simultaneous increase in *T*_*g*_ and substantial decrease in storage modulus presents a complex thermomechanical response that reflects the superposition of multiple competing mechanisms during field service [8], specifically involving residual post-curing, moisture-related plasticization, matrix degradation, and interfacial damage.

**Fig 26:**
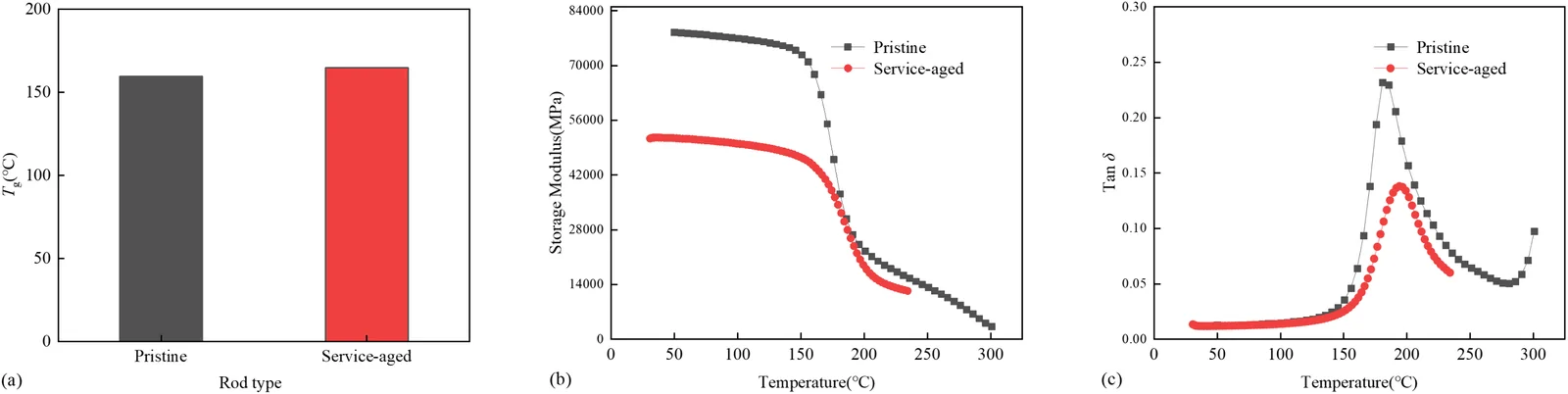
Dynamic mechanical properties of the carbon-fiber core of pristine and service-aged PFRC: (a) glass-transition temperature; (b) storage modulus as a function of temperature; (c) tan *δ* as a function of temperature.

The slight elevation in *T*_*g*_ is consistent with a restriction in the segmental mobility of the polymer network. A plausible mechanism for this localized molecular stiffening is the residual post-curing of unreacted epoxy groups, potentially driven by the downhole thermal environment during the 435-day service period. Because no direct chemical characterization was performed in the present study, the contribution of residual post-curing is explicitly proposed as a hypothesis rather than an experimentally verified mechanism. In a water-rich downhole environment, moisture-related plasticization typically competes with curing by increasing free volume and polymer-chain mobility, which inherently acts to depress *T*_*g*_. The observed net increase in *T*_*g*_ implies that the structural restriction of the polymer chains—hypothesized to result from residual post-curing—may have dominated over the plasticizing effects of the retained moisture at the molecular level.

In contrast, the macroscopic dynamic stiffness of the composite is highly sensitive to physical structural integrity rather than solely to the molecular state of the matrix. The 34.3% reduction in storage modulus and the decreased tan *δ* peak indicate a severe deterioration in dynamic load-bearing capacity and energy-dissipation efficiency. This reduction is consistent with the combined effects of matrix degradation and fiber–matrix interfacial damage. As directly corroborated by the matrix fragmentation and interfacial debonding observed in the SEM analysis, structural degradation physically disrupts the load-transfer pathways. These physical defects mechanically decouple the reinforcing fibers from the surrounding matrix, overshadowing any localized stiffness gains provided by the hypothesized post-curing of the resin. Consequently, the apparent divergence between the elevated *T*_*g*_ and the degraded storage modulus reflects the differing sensitivities of these two parameters to microstructural evolution. *T*_*g*_ is primarily governed by the molecular-level mobility of the polymer network, which was likely influenced by the hypothesized post-curing. Conversely, the dynamic stiffness (storage modulus) is dominated by the physical continuity of the macro-composite structure, which was substantially compromised by matrix degradation and extensive interfacial damage, thereby severely impairing interlaminar stress transfer under dynamic loading.

### 3.4. Moisture desorption analysis

Moisture-desorption behavior was characterized by monitoring the mass loss of pristine and service-aged PFRC specimens during controlled drying. As shown in Fig 27(a–c), the mass-loss ratio increased rapidly during the initial drying stage and subsequently approached a relatively stable equilibrium value. A distinct decrease in the slope of the desorption curves occurred at approximately 14 days, indicating a transition from rapid moisture release to a slower desorption process. Compared with the pristine specimens, the service-aged specimens exhibited consistently higher equilibrium mass-loss ratios in the overall rod, carbon-fiber core, and glass-fiber-rich shell, indicating an increased amount of removable moisture retained within the aged material.

**Fig 27:**
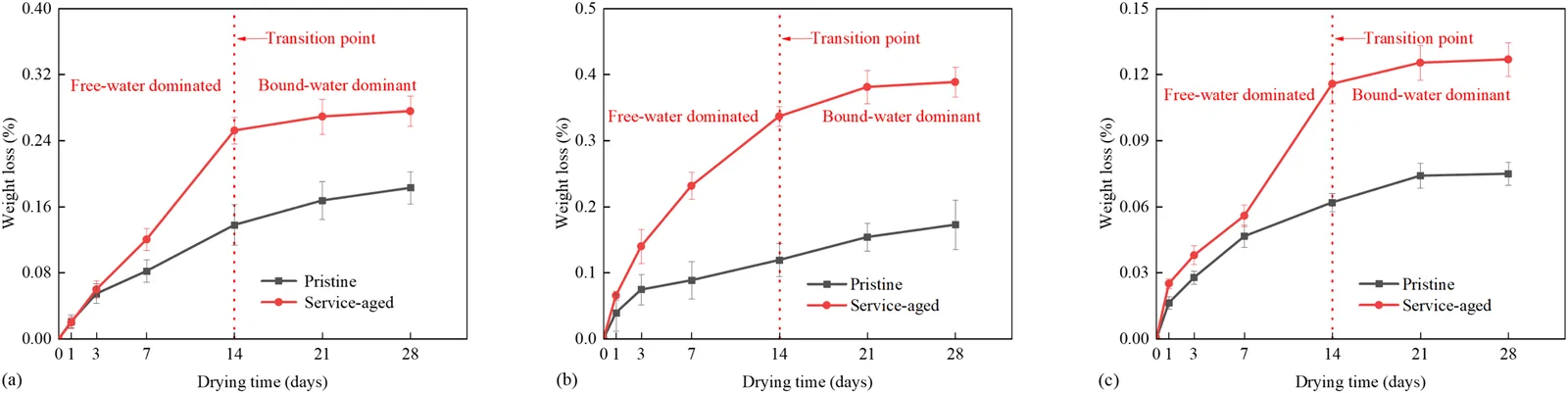
Moisture-desorption curves of pristine and service-aged PFRC: (a) carbon-fiber core; (b) glass-fiber shell; (c) whole PFRC rod.

The higher equilibrium mass-loss ratio primarily reflects the greater quantity of physically or weakly bound moisture that could be released under the adopted drying conditions. Since the present test quantifies drying-induced mass loss, the measured values provide an indirect comparative characterization of retained removable moisture rather than a direct measurement of moisture uptake during downhole service. The observed increase in mass loss therefore reflects a change in moisture-retention behavior after long-term exposure, while the contribution of moisture to chemical modification or mechanical degradation of the polymer matrix cannot be independently quantified from the desorption data.

The magnitude of the increase in equilibrium mass loss varied among the structural regions. The glass-fiber-rich shell exhibited a more pronounced increase than the carbon-fiber core, indicating spatially heterogeneous moisture retention across the radial structure of the PFRC. This difference is consistent with the greater environmental exposure of the outer region and its more extensive damage characteristics observed by SEM, including matrix cracking, resin loss, and fiber–matrix interfacial debonding. Such defects can increase the accessible pathways for moisture ingress and provide additional sites for moisture retention, thereby contributing to the spatial variation in desorption behavior. The correspondence between increased moisture retention and the observed structural damage, however, represents an associated relationship rather than direct evidence of a causal sequence.

The moisture-desorption results, together with the DMA and SEM observations, provide complementary evidence for the evolution of the polymer matrix and fiber–matrix interfaces during long-term service. The greater retained removable moisture in the service-aged material is consistent with a possible contribution of moisture-related plasticization to matrix softening, while the concurrent reduction in storage modulus and the pronounced matrix and interfacial damage observed by SEM indicate a broader deterioration of the matrix-dominated load-transfer structure. The combined results therefore suggest that moisture exposure may have participated in the degradation process, but the relative contributions of moisture retention, matrix degradation, and interfacial damage cannot be quantitatively separated using the present experimental measurements alone.

### 3.5. Damage evolution model of PFRC during long-term downhole service

Based on the mechanical tests, SEM observations, DMA results, moisture-desorption behavior, and field observations, a possible two-phase damage-evolution process comprising five progressive stages is proposed, as illustrated in Fig 28. Because the material was characterized only before and after the 435-day service period without in-situ monitoring, this structural sequence represents a phenomenological reconstruction that integrates experimentally verified damage states with inferred failure mechanisms.

**Fig 28:**
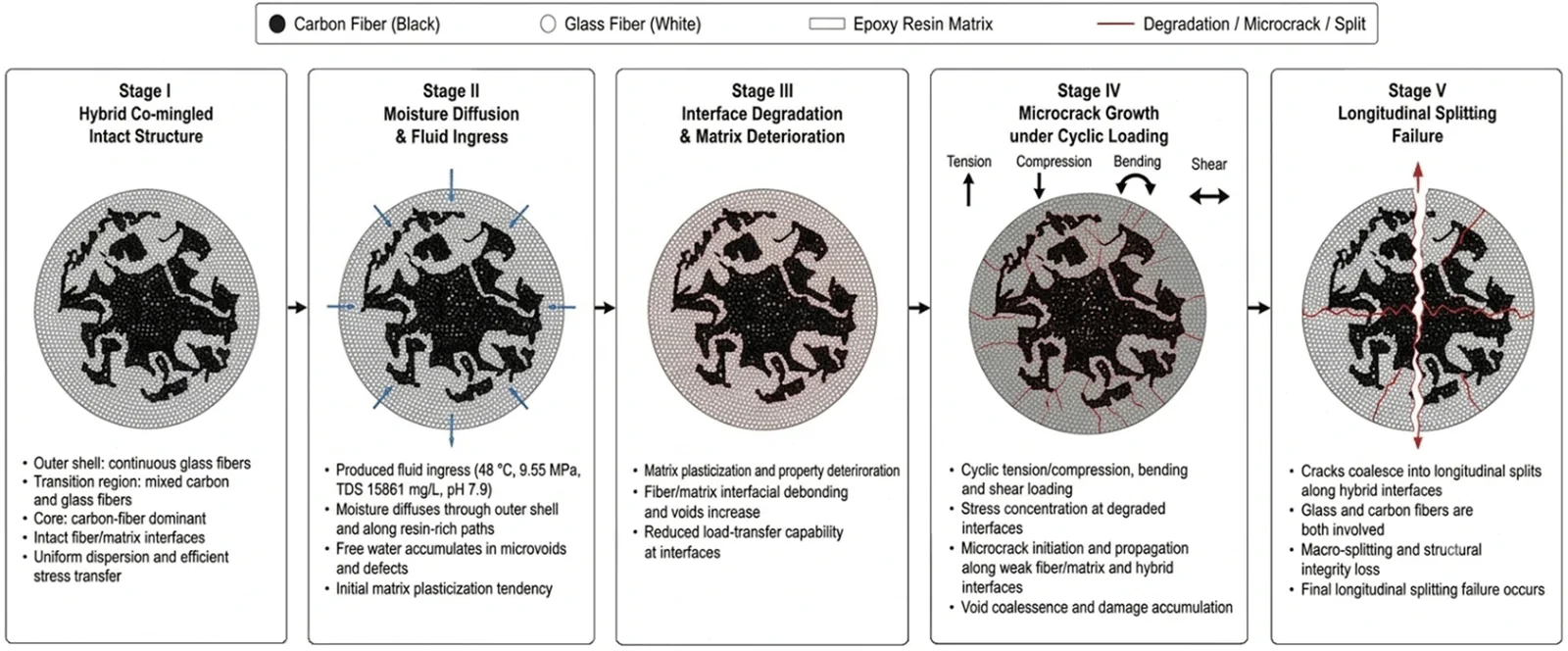
Proposed progressive damage-evolution and splitting model of PFRC during long-term downhole service.

The first phase corresponds to cumulative degradation during downhole service. The directly observed phenomena after field exposure include a 34.3% reduction in storage modulus, greater retained removable moisture in the service-aged PFRC, and extensive matrix fragmentation, resin loss, and interfacial debonding verified by SEM. The mechanical results further confirmed that the properties most dependent on matrix and interfacial stress transfer were more strongly affected: flexural and apparent short-beam shear strengths decreased by 19.92% and 21.98%, respectively, whereas tensile strength decreased by 10.02%. Based on these experimentally confirmed static endpoints, it is logically inferred that prolonged exposure to the water-rich fluid and cyclic axial loading progressively weakened the polymer matrix and the carbon/glass hybrid interfaces over time. While the final deteriorated state of the microstructure is a verified physical fact, the progressive step-by-step matrix softening during downhole operation remains a proposed mechanism.

The second phase corresponds to the retrieval and drum-winding process. It is a documented field observation that the macroscopic longitudinal splitting occurred during retrieval rather than during downhole operation. Microscopic inspection directly confirmed that the carbon/glass hybrid transition region exhibited pronounced interfacial separation, matrix fragmentation, and longitudinal cracking, indicating severe structural vulnerability. During the retrieval process, the rod was subjected to additional bending deformation. Previous experimental and numerical work on similar carbon/glass composite sucker rods has shown that stiffness mismatch between the carbon- and glass-fiber-rich regions can produce deformation incompatibility and elevated interfacial stresses under bending [31]. Therefore, it is inferred that the additional bending imposed during retrieval and drum winding may have promoted the propagation and coalescence of pre-existing interfacial cracks, thereby contributing to the observed local longitudinal splitting. Collectively, while the physical microstructural damage and macroscopic mechanical degradation are experimentally demonstrated, the dynamic propagation of cracks during retrieval and the exact temporal sequence of damage accumulation were not continuously monitored in real time. The proposed model thus describes a plausible, evidence-based sequence of cumulative environmental and cyclic-loading damage followed by bending-assisted crack growth.

## 4. Conclusion

This study evaluated the mechanical degradation and damage evolution of a pultruded carbon/glass-fiber hybrid composite sucker rod after 435 days of downhole service. The mean tensile, flexural, and apparent short-beam shear strengths decreased by 10.02%, 19.92%, and 21.98%, respectively. After Holm correction, the reductions in flexural and apparent short-beam shear strength remained statistically significant (adjusted p < 0.001), whereas the tensile-strength reduction did not meet the 0.05 significance criterion (adjusted p = 0.057). In contrast, the mean compressive strength decreased by only 2.25% and the difference was not statistically significant after Holm correction (adjusted p = 0.800), although its coefficient of variation increased markedly from 2.42% to 18.88%. The Weibull shape-parameter point estimates for tensile, compressive, flexural, and apparent short-beam shear strengths changed from 20.49, 41.31, 37.78, and 39.00 for the pristine PFRC to 13.85, 5.27, 24.07, and 32.10 for the service-aged PFRC, respectively. Given the limited sample size and broad bootstrap uncertainty, these Weibull estimates are interpreted as exploratory descriptors of strength dispersion rather than precise design-level reliability parameters, with the most pronounced increase in experimental strength variability observed under compression. The mean tensile strength of the service-aged PFRC corresponded to a nominal static axial load of approximately 439kN, which remained above the maximum polished-rod load of 94.4kN in the investigated well; however, this comparison does not constitute a formal rod-level reliability or design-code assessment.

In the carbon-fiber core, the storage modulus decreased by 34.3%, while *T*_*g*_ increased from 159.5 to 164.76 °C. Taken together, the DMA, moisture-desorption, and SEM results are consistent with matrix degradation and progressive weakening of the fiber–matrix and internal hybrid interfaces after long-term field service. The greater equilibrium mass loss of the service-aged specimens is consistent with greater retention of removable moisture relative to the pristine baseline, while the slight increase in *T*_*g*_ may reflect residual post-curing. However, these observations do not independently establish the individual chemical mechanisms involved. Matrix fragmentation, resin loss, fiber–matrix debonding, and longitudinal cracking further suggest that the carbon/glass hybrid transition region is particularly susceptible to damage. Accordingly, the proposed damage-evolution model suggests that accumulated matrix and interfacial degradation may have created a preferential pathway for longitudinal crack propagation, while additional bending during retrieval and drum winding may have promoted the propagation and coalescence of pre-existing cracks and thereby contributed to local longitudinal splitting. Because crack evolution was not monitored in situ, this sequence should be regarded as an evidence-based phenomenological interpretation rather than a directly verified temporal process.

These findings demonstrate that long-term performance assessment of hybrid composite sucker rods should consider not only reductions in average strength but also changes in property variability and interface-sensitive performance. Potential mitigation strategies include low-water-uptake and hydrolysis-resistant resin systems, hydrothermally stable fiber-surface treatments and compatible sizing agents, well-dispersed nanofillers, and flexible protective barrier coatings [32–37]. Their effectiveness and manufacturing compatibility should be validated specifically for full-scale PFRC sucker rods under representative downhole conditions.

The conclusions of this study are limited to one well, one rod geometry, a single 435-day exposure duration, and five within-rod subspecimens obtained from one parent rod for each condition rather than independent rod-level replicates. Moreover, the downhole environmental and mechanical loading histories were not continuously monitored. Direct chemical characterization was also unavailable. Future studies should include multiple wells and exposure durations, independent parent rods, larger sample sizes, continuous field monitoring, direct chemical characterization, and quantitative evaluation of the relevant loading and stress conditions through mechanical measurements and validated numerical models.
